# Single-cell landscape of long and short glandular trichomes in *Nicotiana tabacum* leaves

**DOI:** 10.1016/j.isci.2024.110650

**Published:** 2024-08-03

**Authors:** Hongyu Chen, Xiaohan Li, Qing Cheng, Nianmin Shang, Zhijun Tong, Qinjie Chu, Chuyu Ye, Xiner Shen, Qian-Hao Zhu, Bingguang Xiao, Longjiang Fan

**Affiliations:** 1 Institute of Crop Science & Institute of Bioinformatics, Zhejiang University, Hangzhou 310058, China; 2 Beijing Life Science Academy, Changping, Beijing 102209, China; 3 Yunnan Tobacco Agricultural Academy, Kunming 650106, China; 4 CSIRO Agriculture and Food, Canberra, ACT 2601, Australia

**Keywords:** Plant biology, Plant development, Transcriptomics;

## Abstract

Glandular trichomes (GTs) play a crucial role in plant defenses and the synthesis of secondary metabolites. Understanding the developmental trajectory of GTs is essential for unraveling their functional significance and potential applications. Here we established a comprehensive single-cell atlas of *Nicotiana tabacum* leaves, a model plant for GT studies. The atlas included a total of 40,433 cells and successfully captured both long GTs (LGTs) and short GTs (SGTs) from *Nicotiana* leaves. The developmental trajectories of these trichomes were delineated, revealing potential disparities in epidermal development. Comparative analysis of *Arabidopsis* and *Nicotiana* trichome development indicated limited similarity between *Arabidopsis* epidermal non-glandular trichomes and *Nicotiana* LGTs and SGTs, implying the essentiality of studying the genes directly involved in the development of *Nicotiana* GTs for a proper and comprehensive understanding of GT biology. Overall, our results provide profound insights into the developmental intricacies of the specialized GTs.

## Highlights

• Established a single-cell atlas of *Nicotiana tabacum* leaves
• Successfully captured both long and short glandular trichomes
• Developmental trajectories show epidermal development disparities
• *PlanTrichome* database includes this comprehensive cell atlas

## Graphical abstract

**Figure:**
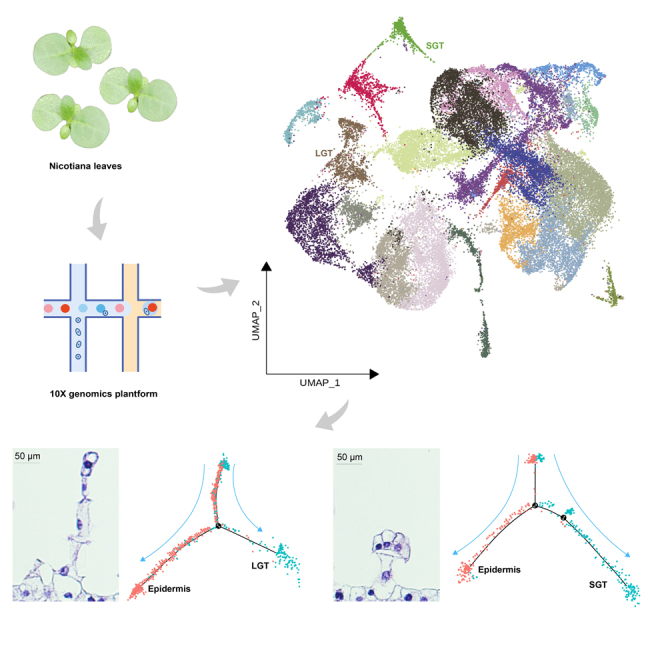

## Introduction

*Nicotiana* species, such as *Nicotiana tabacum* and *N. benthamiana,* have served as model systems for studying various biological processes due to their genetic tractability and amenability to experimental manipulation.^1^^,^^2^^,^^3^^,^^4^ One key characteristic of *Nicotiana* leaves is the presence of glandular trichomes (GTs), specialized epidermal structures that have garnered considerable attention due to their essential roles in plant defense^5^ and secondary metabolite synthesis.^6^^,^^7^ Glandular trichomes are involved in producing a diverse array of secondary metabolites, including alkaloids and terpenoids,^8^ which contribute to the plant’s ability to ward off herbivores,^9^^,^^10^^,^^11^ pathogens,^12^ and environmental stresses.^13^^,^^14^^,^^15^ Glandular trichomes originate from epidermal cells and are found in approximately 30% of vascular plant species.^16^ They exist in various forms and are easily accessible, making them excellent model systems for exploring molecular mechanisms of plant cell differentiation, including cell fate determination, cell cycle control, and cell morphogenesis.^17^

*Nicotiana* plants have two distinct types of glandular trichomes: short-stalk glandular trichomes (SGTs) and long-stalk glandular trichomes (LGTs). SGTs are characterized by a brief, unicellular stalk culminating in a head comprising multiple nonchlorophyllous cells. Conversely, LGTs exhibit a multicellular stalk and a head comprising either a singular or multiple chlorophyllous cells.^18^ Research in the field of *Nicotiana* glandular trichomes can be broadly classified into three main directions. The first avenue centers on comprehending the developmental intricacies of these trichomes. Notable studies have pinpointed pivotal genes and regulatory elements governing trichome initiation, expansion, and maturation. Noteworthy examples include the identification of *NtCycB2*,^19^ *NbGIS*,^20^ *NbJAZ3*,^21^ *NtHD9*, and *NtHD12*^22^ as contributors to trichome development. Another research trajectory pertains to the identification and characterization of trichome-specific metabolic pathways. Renowned for their producing diverse secondary metabolites such as terpenes and phenolic compounds, *Nicotiana* glandular trichomes have spurred investigations into the genes and enzymes responsible for metabolite synthesis, leading to the discovery of their regulatory networks.^23^^,^^24^^,^^25^^,^^26^ The third realm of inquiry involves the exploration of the ecological and agronomic implications of *Nicotiana* glandular trichomes. LGTs play a prominent role in aphid resistance, while SGTs enhance herbivore resistance through the accumulation of defensive proteins. These roles are intimately tied to the gene expression patterns of LGTs and SGTs.^27^ Understanding the mechanisms underlying defense-induced transcriptomic reprogramming and linking the mechanisms to metabolite production holds the potential to inform strategies for effective pest management.

Many studies have delved into the development, metabolism, and ecological significance of *Nicotiana* glandular trichomes. However, our understanding of the differential and development of LGTs and SGTs remains limited due to the technical challenges involved in sampling these two types of glandular trichomes. The most commonly used technique for sampling glandular trichomes is to abrade them from the frozen leaf surface using a brush, followed by mesh filtration to enrich glandular trichomes.^28^^,^^29^^,^^30^ Another method, developed by Gershenzon et al., involves shaking fresh leaves in a viscous liquid medium containing glass beads to detach the glands, followed by filtration to purify them.^31^ Alternatively, tissues are homogenized in a blender, and trichomes are separated from leaf debris through centrifugation with a Percoll density gradient.^32^ All these traditional sampling methods isolate the entire trichome clusters and are unable to distinguish different types of trichomes, which poses challenges in the comprehension of the biology of the different types of trichomes. Single-cell technology that uses protoplasts or nuclei as the starting materials can now be adopted to separate different cell types of a given tissue and to investigate their differentiation and development, allowing for a more accurate understanding of cellular heterogeneity and functional differences among different types of trichomes. By analyzing genes expressed in individual trichome cells, key genes playing a crucial role in trichome development can be identified, leading to a deeper understanding of the mechanisms regulating trichome development. However, achieving comprehensive coverage of all distinct cell types in *Nicotiana* leaves during protoplast preparation presents a significant challenge, primarily due to the inherent difficulties associated with isolating protoplasts from glandular trichomes,^33^ because of their distinctive cellular structure and histological features, such as thicker cell walls.^34^

Here, we utilized an automated instrument to isolate *N. tabacum* leaf cell nuclei, thereby constructing a single-cell atlas of the leaves. This approach provided insights into the developmental dynamics of short and long glandular trichomes and revealed the metabolic diversity among different *N. tabacum* accessions. Furthermore, by integrating our *Nicotiana* data with publicly available single-cell data from *Arabidopsis thaliana*, we elucidated both the similarities and differences between epidermal trichomes in *Nicotiana* and *Arabidopsis*. The identification of cluster-specific or cell-type-specific marker genes represents a valuable resource that significantly advances the field of glandular trichome biology and aids in optimizing metabolite production in pest control strategies.

## Results

### Overview of cellular composition and diversity of *Nicotiana* leaf

**Figure 1:**
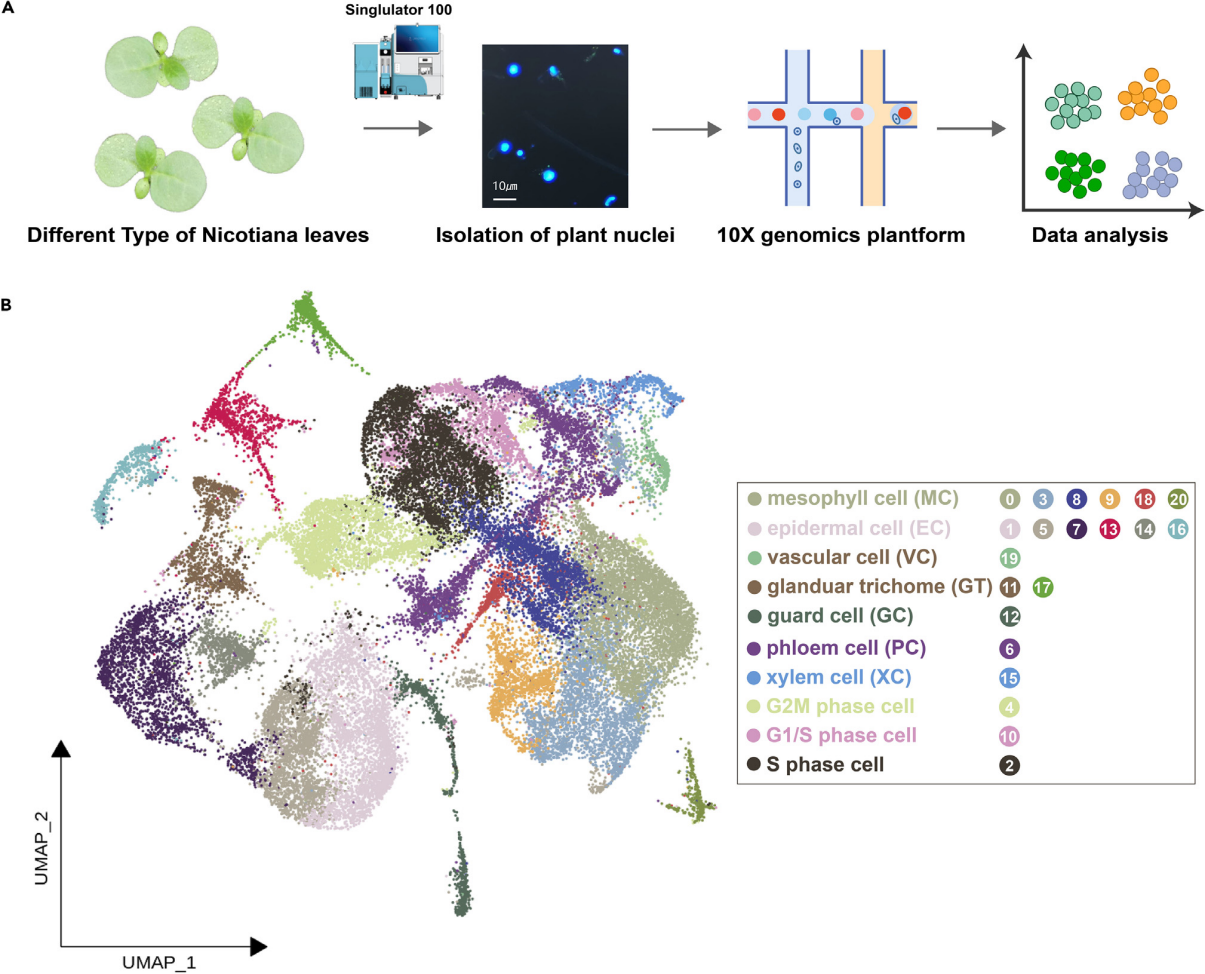
Workflow of this study and single-cell RNA atlas of *Nicotiana* leaves (A) The workflow used by this study in the isolation of single nuclei from young *N. tabacum* leaf, generation and analysis of snRNA-seq data. (B) Visualization of the cell clusters of *Nicotiana* leaves based on their snRNAs by UMAP algorithm. Each dot denotes a single cell. Cell clusters are represented by different colors.

We isolated nuclei from the young leaves of three *N. tabacum* accessions: Basma (highly aromatic oriental), K326 (flue-cured), and B1000-1 (cigar), which represent distinct aromatic properties and metabolic traits (details see the fourth section). To extract nuclei from leaf protoplast cells, we employed an automated device called Singulator-100 (Figure 1A). This apparatus offers precise control over temperature, grinding force, and processing time throughout the ablation process, reducing the risk of variable nuclear fragmentation of different samples caused by temperature and force variations. Following the acquisition of nuclei with a clean background, 10x Genomics transcriptome sequencing was conducted to generate single-cell transcriptomes, which were used in cell-type clustering and downstream analyses (Figure 1A).

A total of six single-cell transcriptomic datasets were generated from the three *Nicotiana* accessions with two replicates per accession. The reference tobacco genome K326 was utilized (details see STAR methods) and each dataset consistently detected a median number of genes exceeding 1,500, indicating the reliability of the data (Figure S1). Apart from adhering to the minimum gene criteria, the cellular data were further refined by setting the threshold for mitochondrial percentage at 0.5%, and for chloroplasts at 5% (Figure S2). Integration of all six single-cell datasets yielded a total of 40,433 cells, which were classified into 21 distinct cellular clusters (Figure 1B). Based on the UMAP plot of each sample, it became evident that each sample exhibited consistency in terms of cell-type clustering. Every cellular cluster was shared across all six samples, underscoring both the robust reproducibility among replicates and the concurrence in the number of distinct cell clusters across the three accessions (Figure S3).

### Annotation of cell types and glandular trichome

To annotate the 21 cellular clusters, we utilized both *Nicotiana* marker genes and homologous marker genes from *A. thaliana* obtained from the PlantscRNAdb database.^35^ Given that *N. tabacum* is an allotetraploid plant, we encountered one-to-many relationships when identifying homologous genes. In this study, we retained homologous genes with alignment scores surpassing the designated threshold (score ≥ 0.7). Based on the presence of *Arabidopsis* marker genes (Table S2), we created scatterplots of marker gene expressions (Figure S4), and eventually, we assigned Clusters 0, 3, 8, 9, 18, and 20 as leaf mesophyll cells, Clusters 1, 5, 7, 13, 14, and 16 as epidermal cells, Clusters 19 and 6 as vascular cells, Cluster 15 as phloem cells, and Cluster 12 as guard cells (Figure 1B). Notably, due to the selection of young leaf tissues, certain cells were undergoing division, and consequently, several clusters containing cells at different division stages were identified. For instance, Cluster 4 was annotated as cells in the mid-G2/M phase, Cluster 10 as cells in the G1/S phase, and Cluster 2 as cells in the S phase. These cells also expressed certain mesophyll cell marker genes, suggesting that they could be mesophyll cells in the midst of division and further experiments are needed to conclusively confirm this hypothesis in the future. Additionally, we collected some experimentally validated tobacco and tomato glandular trichome-related genes. Ultimately, we identified Clusters 11 and 17 as glandular trichome cells (details see next section). Correlation-based analysis of cluster similarity indicated that clusters identified as the same cell type exhibited relatively high correlation (Figure S5), indicating the accuracy of the cell clustering and annotation results. Moreover, despite the inconsistency in their cell numbers, the proportion of each cell type across different samples (genotypes and replicates) was notably consistent (Figure S6).

**Figure 2:**
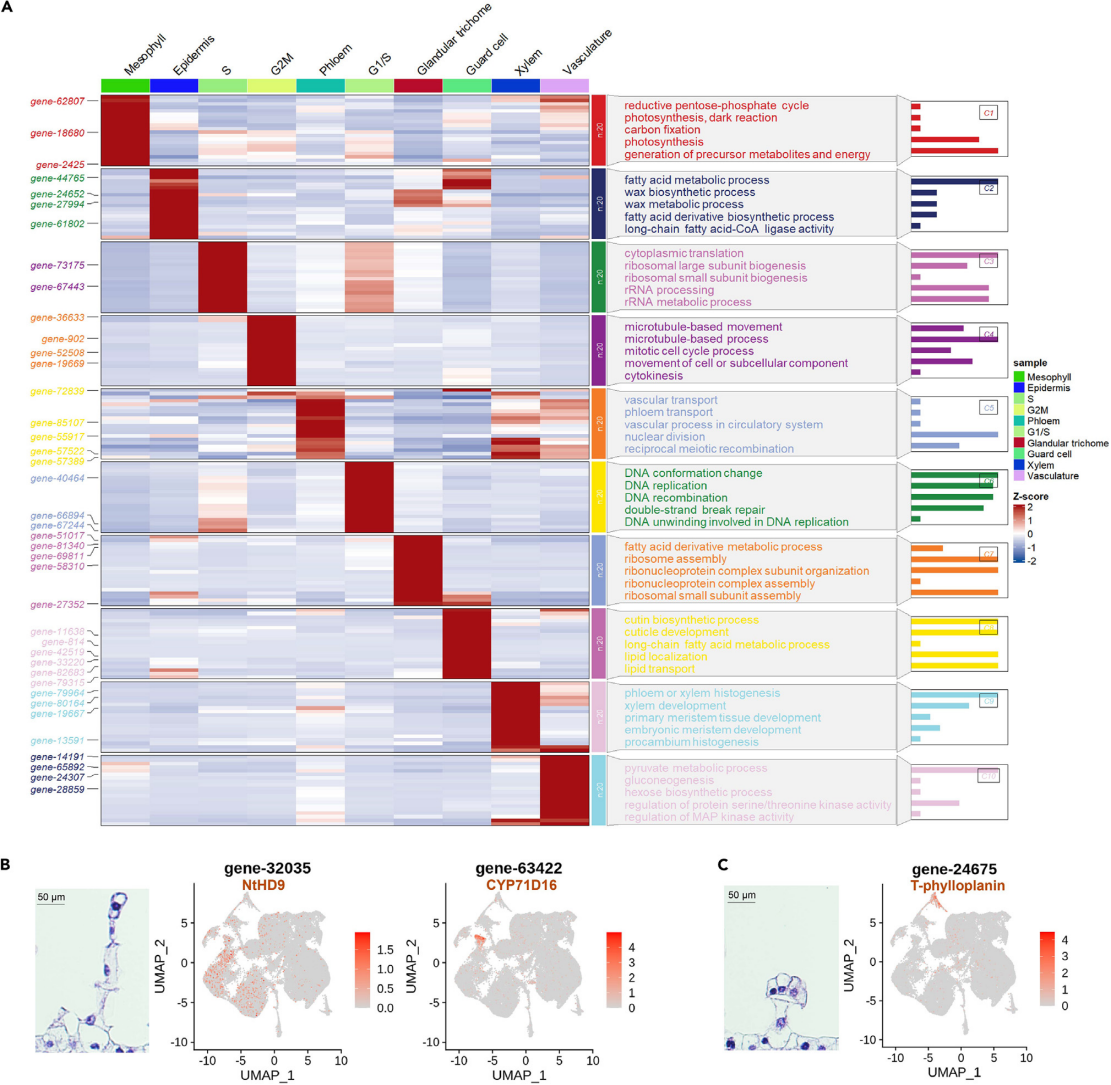
Annotation of cell types and experimental validation of *Nicotiana* glandular trichomes (A) Differential gene expression and functional enrichment in different cell types. The heatmap on the left shows cell-type-specific differentially expressed genes, with the representative enriched functional GO terms displayed in the middle panel. The bar chart on the right panel illustrates the number of enriched genes of the corresponding GO term. (B and C) Known genes specifically expressed in the cell clusters of long (LGTs) and short (SGTs) glandular trichomes, respectively.

To further validate the accuracy of cell type annotations, we identified genes specifically expressed in each cell type and conducted Gene Ontology (GO) pathway enrichment analysis to elucidate their functional characteristics, as presented in Tables S3 and S4. As illustrated in Figure 2A, genes specific to each cell type were enriched with the function of the annotated cell type. For instance, cluster-specifically expressed genes identified in mesophyll cells were enriched in pathways associated with photosynthesis and carbon fixation, which aligns with their involvement in photosynthetic activities. Similarly, cluster-specifically expressed genes enriched in cells undergoing the G2/M phase were linked to biological processes such as microtubule-based activities and the mitotic cell cycle, in line with the identity of the cells of the cell-cycle phase (Table S4). These results provide further support evidence for the reliability of the cell-type annotation.

Two clusters (11 and 17) were annotated as glandular trichome cells. To verify their identities, we examined the expression of genes specifically associated with either LGTs or SGTs. We found expression of two LGT-specific genes *NtHD9*^22^ and *CYP71D16*^36^ in Cluster 11 but not in Cluster 17 (Figure 2B). Interestingly, the LGT developmental gene *NtHD9* is predominantly expressed in the lower portion of the Cluster 11 clade, while the metabolic synthesis-related gene *CYP71D16* shows predominant expression in the upper part of the Cluster 11 clade. Additionally, T-Phylloplanin, a potential antimicrobial compound found on the surface of certain plants, was previously identified to be secreted by SGTs in *N. tabacum*.^37^ We found the gene encoding T-Phylloplanin being expressed exclusively in Cluster 17 (Figure 2C). These results enabled us to define Cluster 11 as LGTs and Cluster 17 as SGTs. Additionally, for further validation, we used the marker genes of *Solanaceae* trichomes from the tomato plant, *SlCycB2* and *SlARF3* (Feng et al., 2021). Intriguingly, *SlCycB2* showed specific expression in the LGTs while *SlARF3* expressed specifically in the SGTs (Figure S7). Further, GO enrichment analysis of the genes specific to LGTs or SGTs revealed that the genes in Cluster 11 (LGTs) were predominantly associated with photosynthesis, consistent with the description of LGTs containing chloroplasts,^38^ and that the genes of Cluster 17 (SGTs) encompassed pathways associated with differentiation, such as “abaxial cell fate specification” (Figure S8, Tables S5, and S6).

We further selected the top three newly identified DEGs in LGTs or SGTs to experimentally validate their cell-type specificity. UMAP visualization revealed that these genes exhibited specific expression patterns within their corresponding cell clusters (Figure S9). *In situ* hybridization experiments were conducted using *Nicotiana* leaf sections from the same developmental stage confirmed the specific expression of gene-65106 (fatty acid reductase 4), gene-10705 (major latex-like protein), and gene-37943 (protodermal factor 1) in LGTs, and the specific expression of gene-35261 (pollen Ole e 1 allergen and ontribut family protein), gene-50725 (phosphoenolpyruvate carboxykinase), and gene-67244 (phosphoenolpyruvate carboxykinase) in SGTs (Figures S9 and S10). These genes could be used as LGTs and SGT markers in future studies.

### Different developmental trajectories for long glandular trichomes and short glandular trichomes cells

**Figure 3:**
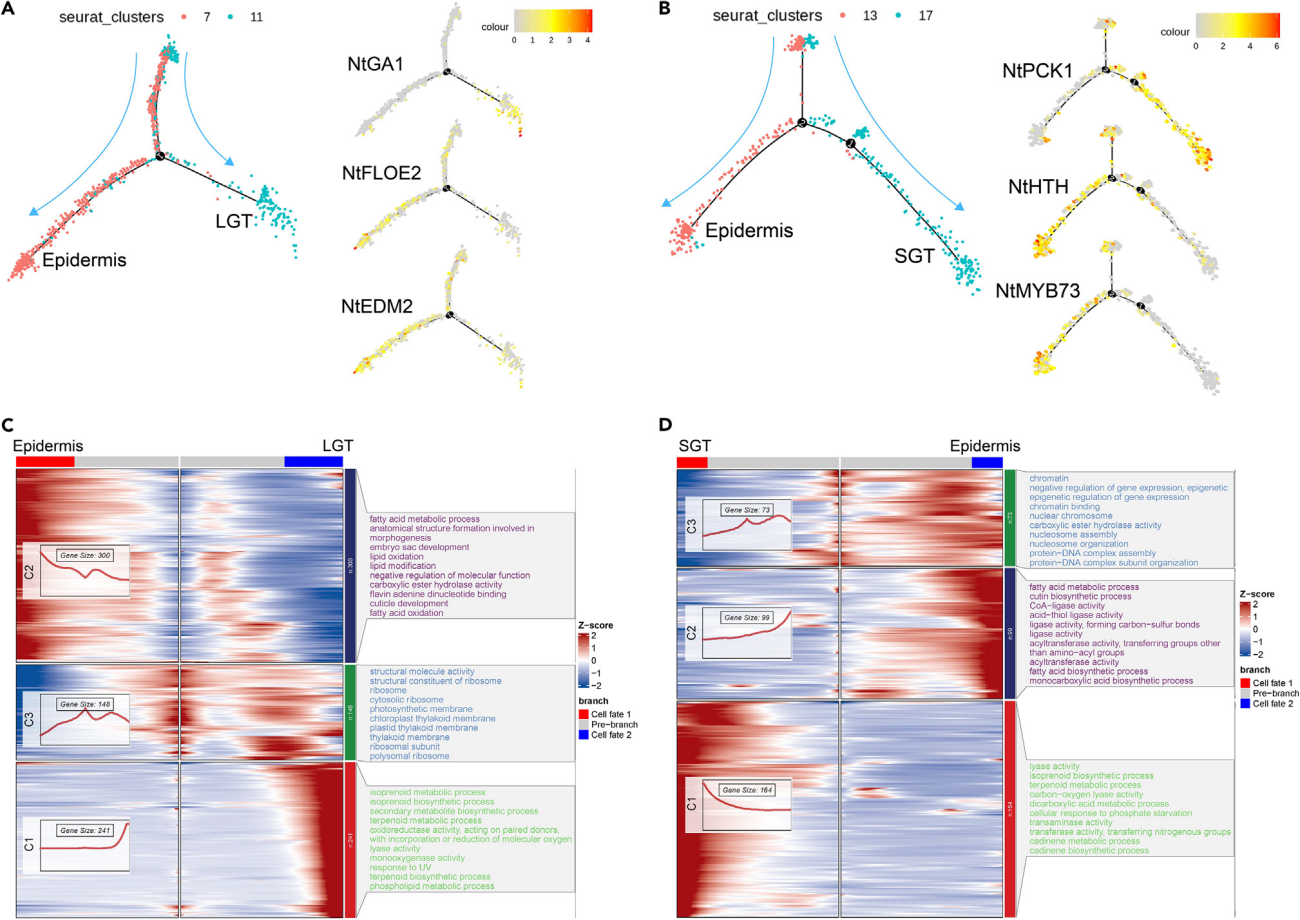
Developmental trajectory analysis of LGT and SGT cells in *Nicotiana* leaf (A) UMAP visualization of LGT developmental trajectory, with expression profiles of development-related genes presented on the right. (B) UMAP visualization of SGT developmental trajectory, with expression profiles of development-related genes presented on the right. (C) Heatmap displaying the expression profiles of differentially expressed genes at branchpoint 1 of the LGTs developmental trajectory, with the corresponding results of Gene Ontology enrichment results showing on the right. (D) Heatmap illustrating the expression profiles of differentially expressed genes at branchpoint 2 of the SGT's developmental trajectory, with the corresponding results of Gene Ontology enrichment results showing on the right.

Subsequently, we constructed developmental trajectories of LGTs, SGTs, and their corresponding epidermal cells. Remarkably, both LGTs and SGTs displayed distinct branching trajectories (Figures 3A and 3B). Importantly, LGTs and SGTs shared a common starting point with epidermal cells, before diverging into separate branches. At the branching point of LGTs (Cluster 11), *NtGA1*, a gene encoding *ent*-kaurene synthase A (KSA) which catalyzes the first step of the gibberellin biosynthetic pathway,^39^ was prominently expressed. Conversely, *NtFLOE2* and *NtEDM2* were predominantly expressed in the epidermal cell branch (Figure 3A). Similarly, in the developmental trajectory of SGTs, cell-type marker genes also showed specific expression within distinct branches. For instance, *NtPCK1* was prominently expressed in the SGTs branch, with its expression gradually increasing along the developmental trajectory. Meanwhile, *NtHTH* and *NtMYB73* were mainly expressed in the epidermal cell branch (Figure 3B).

Furthermore, we conducted Gene Ontology (GO) enrichment analysis for the genes differentially expressed at branching point 1 of the LGTs trajectory and the branching point 2 of the SGTs trajectory and further clustered them based on their expression profile during the developmental trajectory (Tables S7, and S8). The DEGs in the LGTs trajectory could be separated into three clusters. Cluster C1 contained 241 genes exhibiting LGT-specific expression, significantly enriched with GO terms related to the isoprenoid metabolic process, secondary metabolite biosynthetic process, and terpenoid metabolic process. Cluster C2 comprised 300 genes primarily displaying epidermal-specific expression, with enriched GO terms associated with distinct epidermal characteristics, such as fatty acid metabolic process, lipid oxidation, and cuticle development. Cluster C3 consisted of 148 genes specifically expressed at the branching point, playing a critical role in the determination of the LGTs’ identity of cells. These genes were mainly associated with cell structure and biosynthetic processes, including “structural molecule activity,” “structural constituent of ribosome,” “ribosome,” “cytosolic ribosome,” and “thylakoid membrane” (Figure 3C). Similarly, the DEGs of the SGT's trajectory were also clustered into three groups. Notably, the genes in Cluster C3 were mainly expressed at the branching point, which is a critical time point in determining the identification of SGTs. The 73 genes in Cluster C3 were enriched with GO terms related to the cell cycle, including “chromatin binding,” “nuclear chromosome,” and “nucleosome assembly.” Similar to the epidermal (C2) and LGTs (C1) cluster in the LGTs trajectory, the epidermal cluster (C2) in the SGTs trajectory contained genes enriched with GO terms such as fatty acid metabolic process and cutin biosynthetic process. Additionally, the SGT's cluster (C1) genes were enriched with GO terms related to terpenoid biosynthesis and metabolism, particularly including the synthesis and metabolism of cadinene (Figure 3D). Taken together, these analyses identified genes playing key roles in the determination of LGTs and SGTs and laid the foundation for further functional characterization of these key genes in the development of LGTs and SGTs.

### Differential metabolic activities of long glandular trichomes and short glandular trichomes cells in different *N. tabacum* accessions

**Figure 4:**
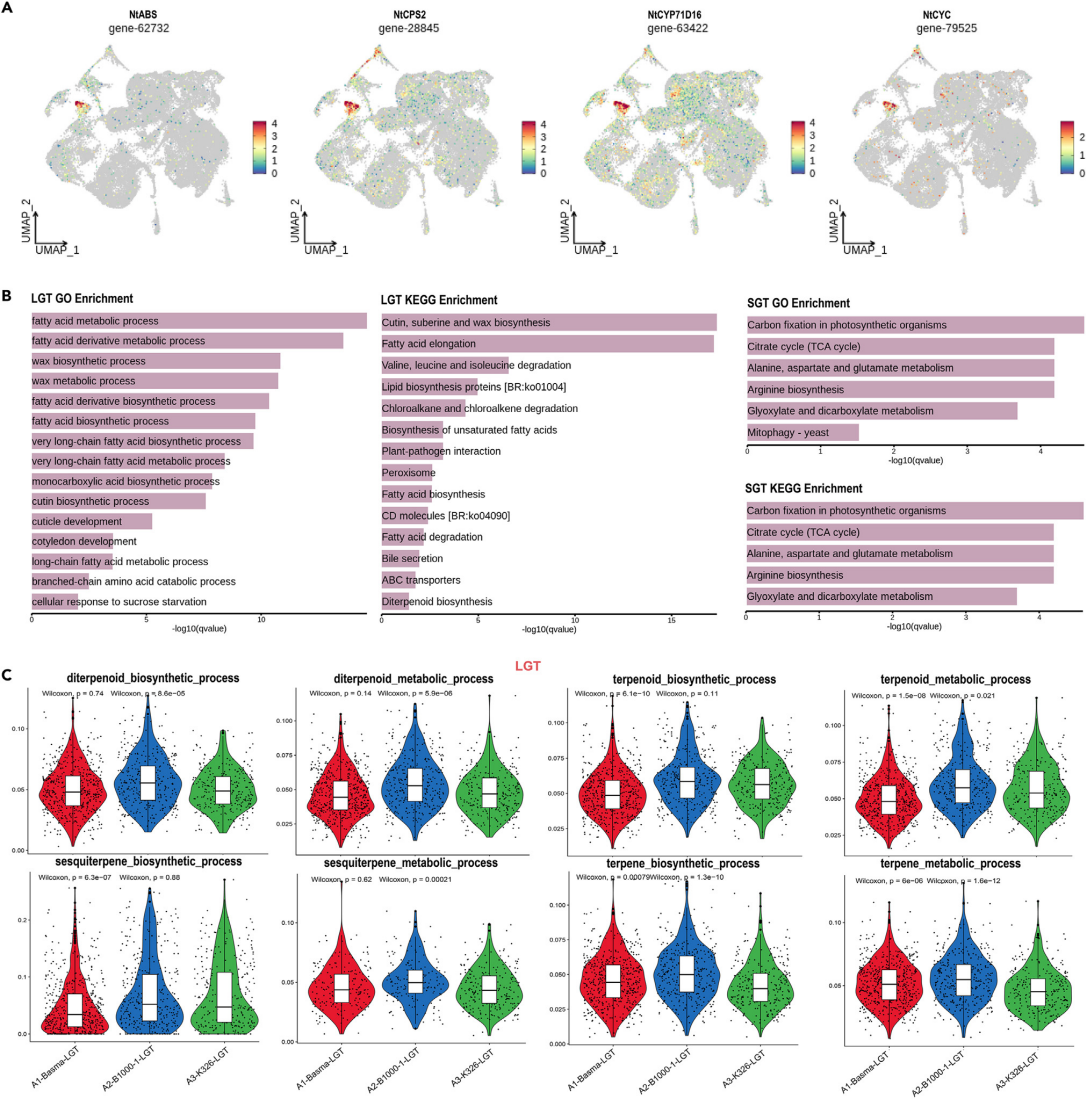
Comparative metabolic activity of long and short glandular leaf trichomes among different *N tabacum* accessions (A) UMAP plots illustrate the expression profiles of labdanoid synthesis-related genes and cembranoid synthesis-related genes. (B) Results of GO and KEGG enrichment analyses for differentially expressed genes in LGTs and SGTs. (C) Violin plots illustrating biosynthetic activity of cembranoid-related pathways in LGTs of three different *N. tabacum* accessions.

The acquisition of long and short glandular trichome cell clusters opens up possibilities for further research on gene expression related to glandular trichomes. Previous studies have identified two types of diterpenes, cembranoids and labdanoids in *N. tabacum*.^40^ Investigating these metabolites at the single-cell level may offer distinct advantages. We firstly examined the expression patterns of four labdanoid and cembranoid synthesis-related genes (*NtABS* and *NtCPS2* for labdanoid synthesis; *NtCYC* and *NtCYP71D16* for cembranoid synthesis) in the *Nicotiana* leaf trichome transcriptome. Intriguingly, these genes exhibited exclusive expression within glandular trichomes, primarily in the LGTs (Figure 4A) The results demonstrate a similar pattern with more metabolic-related genes, which suggested that LGTs potentially encompass some non-glandular trichomes at an early developmental stage and not yet engaged in metabolic processes. Given the developmental stage of the *Nicotiana* leaf tissue used in our single-cell sequencing study and the recognized pattern of development between short and long glandular trichomes,^41^ it is reasonable to speculate that the SGTs, demonstrating a lower expression level, might still be in their developmental phase. Directly following this, we used the FindConservedMarkers method to contrast the expression patterns between long and short glandular trichomes and identify consistently marked genes across various *N. tabacum* accessions. This led us to perform GO and KEGG enrichment analyses. The subsequent findings illustrated that, in the GO enrichment analysis, SGTs were predominantly linked with various developmental pathways. Meanwhile, in the KEGG enrichment analysis, they were primarily engaged in energy metabolism pathways, showing a less pronounced presence in terpenoid or fatty acid metabolism-related pathways. On the other hand, LGTs showed enrichment in numerous pathways captured in both GO and KEGG analyses, particularly in the KEGG outcomes where diterpenoid biosynthesis had a significant representation (Figure 4B). This information further corroborates previous findings which suggest that the glandular trichomes on *Nicotiana* leaves play a significant role in secreting a variety of vital biochemical compounds, encompassing sucrose esters, waxes, micro-elements, and diterpenoids.^42^ Our research has brought these observations into sharper focus by pinpointing that these key compounds are primarily expressed in the long glandular trichomes of tobacco.

Subsequently, we compared the pathway activity expressions of cembranoid-related metabolic processes in three distinct *N. tabacum* accessions (B1000-1, Basma, and K326) (Figure 4C). Basma is renowned for its rich aroma and is characterized by a complex mixture of aromatic compounds; K326 falls into the category of flue-cured *N. tabacum* and is commonly utilized in the traditional cigarette industry while B1000-1 displays a unique array of aromatic compounds specifically for cigar production. Notably, B1000-1 exhibited significantly higher pathway activities, consistent with previous findings obtained through gas chromatography-mass spectrometry profiles.^43^ In contrast, Basma and K326 exhibited comparable levels of pathway activity. Therefore, the application of single-cell technology allowed us to achieve a finer-grained comparison among the *N. tabacum* accessions, revealing potential genes responsible for the differences. Furthermore, we identified genes exhibiting differential expression between K326 and others in LGTs and SGTs (Tables S9 and S10). In LGTs, Basma displayed the highest number of upregulated DEGs, while B1000-1 exhibited the highest number of downregulated DEGs. Conversely, in SGTs, B1000-1 displayed the highest abundance of DEGs (Figure S11). These DEGs represent valuable resources for subsequent research aimed at elucidating the potential mechanisms underlying differences in secondary metabolite production.

### Distinct development of *Arabidopsis* and *Nicotiana* glandular trichomes

**Figure 5:**
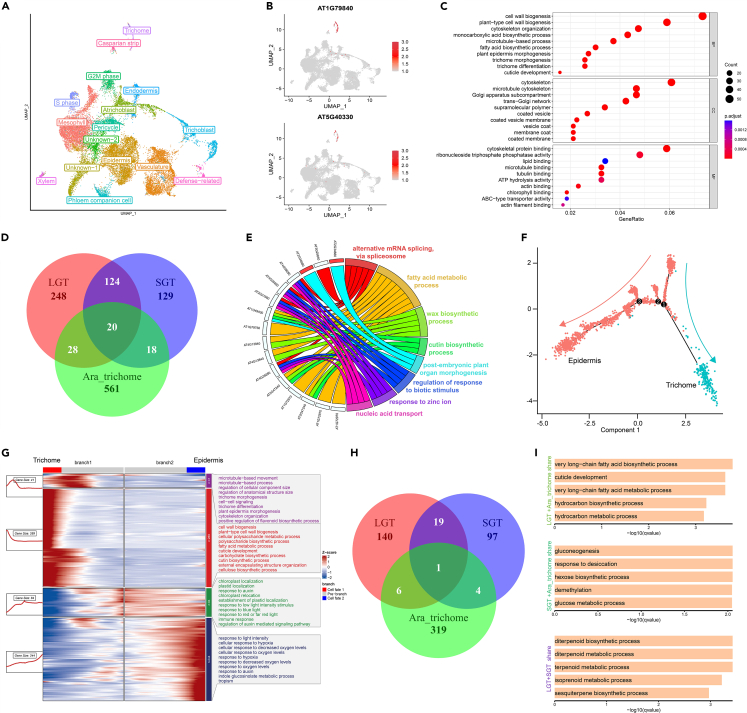
Comparison of epidermal trichome development between *Arabidopsis* and *Nicotiana* (A) UMAP plot depicting clustering of cell types in 6-day-old *Arabidopsis* seedlings. (B) UMAP plot illustrating the specific expression pattern of epidermal trichome marker genes in *Arabidopsis*. (C) Dot plot displaying the Gene Ontology (GO) enrichment results of cluster-specific genes in *Arabidopsis* epidermal trichomes. (D) Venn diagram revealing the intersection of cluster-specific genes expressed in *Arabidopsis* epidermal trichomes and *Nicotiana* LGTs and SGTs. (E) Major GO pathways enriched in the 20 genes overlapping between the *Arabidopsis* trichomes and the two types of *Nicotiana* trichomes. (F) Developmental trajectory plot of *Arabidopsis* epidermal cells and trichomes, with red points representing epidermal cells and blue points representing trichome cells. (G) Heatmap showing the expression profile of differentially expressed genes at branching point 1 of the developmental trajectory of *Arabidopsis* epidermal cells and trichomes. The numbers and the lines in the left boxes represent the number of genes and the gene expression patterns, respectively. The right boxes display enriched GO terms of the differentially expressed genes. (H) Venn diagram illustrating the overlap of genes specifically expressed in *Arabidopsis* trichomes development and *Nicotiana* LGTs and SGTs. (I) GO terms enriched in the intersecting genes showing in (H), from top to bottom: genes overlapping between LGTs and *Arabidopsis* trichomes, between SGTs and *Arabidopsis* trichomes, and between LGTs and SGTs.

Unlike the *Nicotiana* leaf epidermal trichome, the *A*. *thaliana* leaf epidermal trichome represents a non-secreting unicellular structure that is frequently adopted as a model system for investigating plant trichome initiation and development.^44^^,^^45^ Therefore, it is of interest to know the trajectory similarities and differences of the two types of trichomes. Given that trichomes on the *Arabidopsis* epidermis initiate in the shoot apical meristem at a very early stage,^46^^,^^47^ we acquired a publicly available single-cell dataset generated from 6-day-old *Arabidopsis* seedlings^48^ and used it in the comparative analysis. We conducted cell clustering and cell-type annotation of the *Arabidopsis* dataset using the same pipeline applied in the *Nicotiana* datasets. The cells were separated into 16 clusters. Except for the two “Unknown” clusters and the “Defense-related” cluster, all of the remaining 13 clusters were representatives of the cell types present in the seedlings (Figure 5A). Notably, the characteristic trichome marker genes AT1G79840 (*GL2*^49^^,^^50^) and AT5G40330 (*MYB23*^51^) were specific to the trichome cluster (Figure 5B). Additionally, for the annotation of *Arabidopsis* trichomes, an independent transcriptomic dataset related to *Arabidopsis* trichomes by the microarray platform^52^ was used and the *Arabidopsis* trichome-specific highly expressed genes identified by us indeed highly expressed in trichomes (Figure S12). The outcomes further confirm our annotation results. In order to corroborate the accuracy of the cell-type annotation results, we also identified cell-type-specific genes and subjected them to GO term enrichment analysis. As illustrated in Figure S13A, the enriched GO terms of each cluster were in line with the annotated cell types. For instance, the cluster representing pavement cells displayed enrichment of marker genes associated with biological processes such as “photosynthesis,” “photosynthesis, light reaction,” and “photosynthesis light harvesting in photosystem I.” Meanwhile, the cluster annotated as epidermal trichomes was enriched with the “fatty acid derivative biosynthetic process” and “cutin biosynthetic process.” Delving deeper into the genes associated with the enriched GO terms in epidermal trichomes, we noticed the presence of genes known to function in trichome development, such as AT5G23940 (*PEL3*^52^^,^^53^), AT1G19835 (*TCS1*^54^), and AT5G40330 (*MYB23*^51^) (Figure S13B), and observed a clear enrichment of biological process associated with trichome development, such as “trichome morphogenesis,” “trichome differentiation,” and “trichome branching” (Figure 5C).

Comparing the genes specific to *Arabidopsis* epidermal trichomes to those specific to *Nicotiana* LGTs or SGTs, we found that most genes were unique to each type of trichome, with 48 genes shared by *Arabidopsis* trichomes with *Nicotiana* LGTs, and 38 genes shared by *Arabidopsis* trichomes with *Nicotiana* SGTs, and 144 genes shared by *Nicotiana* LGTs and SGTs. Of these genes, only 20 were found in all three types of trichomes (Figures 5D and Table S11). These results suggest that, while the development of non-secreting and secreting trichomes is largely determined by different sets of genes, a small set of genes could be fundamental for the development of both types of trichomes. The 48 genes common in *Arabidopsis* epidermal trichomes and *Nicotiana* LGTs, were enriched with GO terms such as “cuticle development” and “wax biosynthetic process,” and GO terms specifically related to epidermal differentiation, such as “epidermis development” and “epidermis cell differentiation.” Among the 48 genes, some have been functionally validated in both *Arabidopsis* and *Nicotiana* trichomes, such as AT4G04890 (*PDF2*) (Figure S14A).^55^^,^^56^^,^^57^ The 38 genes exclusively enriched in *Arabidopsis* epidermal trichomes and *Nicotiana* SGTs were found to be associated with GO terms “cytoskeleton organization,” “mitotic cell cycle,” and “plant-type cell wall biogenesis,” implying a degree of similarity in terms of cell cycle and cell wall synthesis (Figure S14B). Few studies have been conducted on SGTs in *Nicotiana* and other plants, therefore, the 38 genes provide a unique opportunity for the investigation of the commonality between non-secreting trichomes and SGTs in the future. The 20 genes common in the three types of trichomes were predominantly related to plant leaf epidermis, enriched with GO terms “fatty acid metabolic process,” “wax biosynthetic process,” “cutin biosynthetic process,” and “regulation of response to biotic stimulus” (Figure 5E).

In order to explore the similarities and differences between *Arabidopsis* trichomes and *Nicotiana* glandular trichomes from a developmental perspective, we conducted a comparative trajectory analysis of *Arabidopsis* trichomes and epidermal cells (Figure 5F). By identifying DEGs at the branching point 1, we obtained genes associated with trichome development (Figures 5G and Table S12). Among them, 84 genes were found to exhibit differential expression at this branching point, primarily enriched in processes related to energy production and growth regulation, such as chloroplast localization, plastid localization, response to auxin, and response to low-light-intensity stimulus. The genes associated specifically with trichome development were categorized into two groups. The first group with 41 genes was expressed during the early to mid-stage of trichome development and significantly related to trichome differentiation. The enriched GO terms of these genes included microtubule-based movement, microtubule-based process, regulation of cellular component size, trichome morphogenesis, and trichome differentiation. The second group with 289 genes was expressed during the later stage of trichome development, mainly associated with trichome maturation and cell wall synthesis, including cell wall biogenesis, plant-type cell wall biogenesis, and fatty acid metabolic process (Figure 5G). Furthermore, we compared the genes related to the development of *Arabidopsis* trichomes and *Nicotiana* LGTs/SGTs. Only one gene was found to be shared by the three types of trichomes, reflecting the specificity of gene expression among the different types of trichomes (Figure 5H). In terms of pairwise comparison, the genes shared between *Arabidopsis* trichomes and *Nicotiana* LGTs were primarily enriched with GO terms closely associated with plant epidermal structure, function, and adaptation to the environment, such as very long-chain fatty acid biosynthetic process, cuticle development, and hydrocarbon biosynthetic process. Notably, in *Arabidopsis*, these pathways were mainly enriched during the later stage of trichome development (Figure 5G). The genes shared between *Arabidopsis* trichomes and *Nicotiana* SGTs were primarily enriched in energy metabolism and response to environmental stress processes, including gluconeogenesis, response to desiccation, and hexose biosynthetic process. The genes shared between LGTs and SGTs were mainly related to terpene synthesis, encompassing the abundant diterpenoids, terpenoids, isoprenoids, and sesquiterpenes (Figure 5I).

Taken together, it becomes evident that while there is a certain degree of overlap among the genes contributing to the development of *Arabidopsis* and *Nicotiana* epidermal trichomes, more genes are unique to *Arabidopsis* trichomes or *Nicotiana* glandular trichomes, which could be the keys for understanding the mechanisms underlying the differential development of different types of plant glandular trichomes.

### An online platform for cell atlas of plant glandular trichome

**Figure 6:**
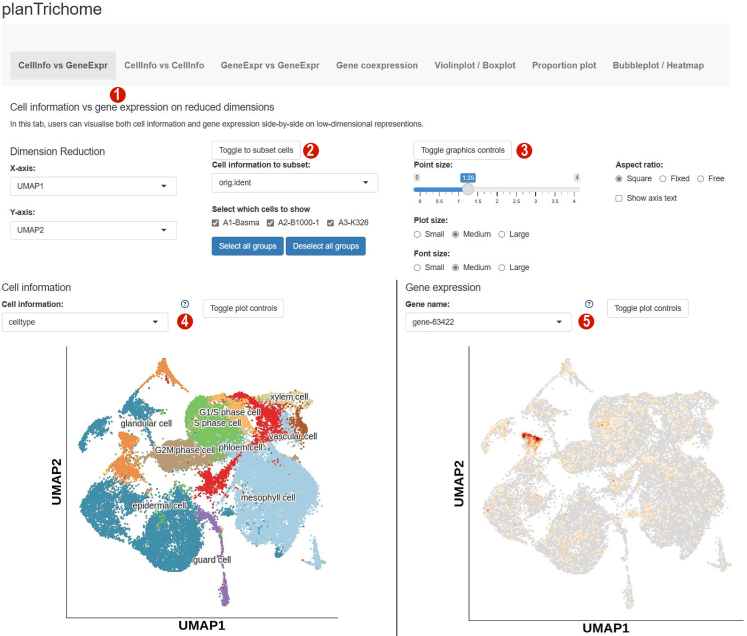
The overview of planTrichome, a single-cell atlas platform of plant trichomes This screenshot illustrates the key features of the online database of plant trichome single-cell atlas (http://ibi.zju.edu.cn/planTrichome/). Numbers on the red circle background represent distinct operational modules.

To advance our comprehension of cellular diversity within leaves, we have established a comprehensive single-cell atlas database of plant trichomes named planTrichome (http://ibi.zju.edu.cn/planTrichome/). This platform serves as a centralized hub for accessing and exploring the intricate cellular composition of trichomes and their gene expression patterns within plant leaves. The platform comprises seven distinct visualization modules (Figure 6). Notably, the “CellInfo vs. GeneExpr” module employs dimensionality reduction plots to visualize cellular clustering information alongside individual gene expression profiles. This is exemplified by panels 4 (cell information) and 5 (gene expression) in Figure 6, where panel 4 showcases leaf cell cluster annotations and panel 5 illustrates the specific expression of gene-53422 in LGTs. The dimensionality reduction plots can be customized with various coordinate systems such as UMAP, t-SNE, and PCA. Given the inclusion of diverse *N. tabacum* accessions in our dataset, users seeking to focus on a specific accession or sample can extract and display the relevant information using tailored parameters, as indicated in panel 2 (Figure 6). Panel 3 outlines the specific parameters controlling the display of the control image. The remaining six visualization modules on the top menu tab follow a similar structure. In particular, the “CellInfo vs. CellInfo” and “GeneExpr vs. GeneExpr” tabs offer a side-by-side comparison of cell information or gene expression, respectively. The “Gene Coexpression” module amalgamates the gene expression of two genes onto a dimension-reduced plot, with varying color hues indicating expression levels, and the number of cells expressing either or both genes being provided. “Violinplot/Boxplot” depicts the distribution of continuous cell information, such as nUMI or module scores, and gene expression across clusters or groups through violin plots or boxplots. The “Proportion Plot” module portrays the composition of different cell clusters or groups using proportion plots, allowing users to plot either cell numbers or proportions. Lastly, the “Bubbleplot/Heatmap” module enables the visualization of the expression of multiple genes across clusters or groups using bubble plots or heatmaps, with the ability to further hierarchically cluster genes and groups.

## Discussion

This study presents a comprehensive transcriptomic analysis of *Nicotiana* leaf tissues at single-cell resolution with a focus on glandular trichomes (GTs). The results contribute to a holistic understanding of glandular trichome biology, offering new insights into the trajectory of GTs and new directions for future research.

In addition to constructing a single-cell atlas of *Nicotiana* leaves, a significant contribution of this study lies in the identification of GT clusters and the establishment of developmental trajectories for LGTs and SGTs, offering a fresh perspective on their morphogenesis. GTs in plants are epidermal outgrowths endowed with the capacity to biosynthesize and secrete specialized metabolites, holding profound scientific and practical significance. Our current understanding of the process of GT development remains limited, largely due to experimental constraints in isolating individual trichome cells for in-depth study. Traditional research on GTs has heavily relied on comparative studies with homologous genes sourced from other species, such as *Arabidopsis* and tomato.^58^ For instance, a recent study on *N. benthamiana* cloned *NbCycB2* and *NbWo*, homologs of *SlCycB2* and *SlWo*, respectively, which have been demonstrated to be involved in trichome development in tomatoes. Detailed gene interaction investigation unveiled that *NbCycB2* and *NbWo* achieve their role in GT development through a novel reciprocal regulatory mechanism.^55^ In this study, we took a genome-wide approach to investigate the genes contributing to GT initiation and development at a single-cell resolution, aiming to have a better grasp of the coordinated events shaping the unique leaf epidermal structures and enhancing our comprehension of GT biology. The cluster-specifically expressed genes in LGTs and SGTs serve as a valuable resource for subsequent functional investigations. Furthermore, by examining the expression patterns of the genes associated with the biosynthesis of the metabolites potentially accumulated in GTs at the single-cell transcriptome level, we discovered that they were accession-specific, implying an intrinsic association of GT composition with metabolites. Regrettably, in this study, we did not explicitly define the presence of fewer non-glandular trichomes in tobacco leaves. However, we observed that Cluster 11 also encompasses numerous cells with relatively lower activity in synthesizing substances such as terpenes. Concurrently, existing research has indicated an inherent morphological relationship between tobacco glandular and non-glandular trichomes, suggesting the potential for tobacco non-glandular trichomes to transition into long-stalked glandular trichomes.^22^ Therefore, Cluster 11 might contain a mixture of non-glandular trichome cells. Further in-depth investigations are warranted for subsequent studies. Moreover, the application of single-cell transcriptomics in conjunction with single-nuclei extraction techniques for studying trichome development showcased in this work can serve as a valuable model for other species, such as tomato which has eight types of leaf trichomes, including four types of glandular trichomes.^9^ However, this study also has its limitations, such as the temporal sampling constraints in the metabolic analysis of short glandular trichomes. Future research endeavors may benefit from incorporating a more comprehensive time-course sampling approach, specifically tailored to address the unique metabolic activities of short glandular trichomes. This would help rectify the current gaps in our understanding of short glandular trichome biology.

This study also presents, for the first time, a comparative analysis of epidermal trichome development in *Arabidopsis* and *Nicotiana* at the single-cell resolution. Previous investigations into the trichome morphology and development often relied on homologous genes from model species such as *Arabidopsis*. However, due to significant species-specific differences, this approach introduced considerable uncertainty. Notably, many plants possess multicellular trichomes, whereas *Arabidopsis* has single-cell non-glandular trichomes. These distinctions encompass differences in cell composition, developmental processes, and metabolism.^59^ For instance, overexpressing the GL1 gene had no impact on trichome formation in *Nicotiana* plants,^60^ indicating different molecular mechanisms governing non-glandular trichome (NGT) and glandular trichome development.^19^ This variation may be attributed to distinct growth conditions, and the adaptive pressures experienced by different plant species, subsequently influencing the attributes and functions of their respective trichomes. These disparities could encompass alterations in the gene expression profiles, expansion or contraction of functional genes, and modifications in regulatory pathways.^45^^,^^61^^,^^62^^,^^63^ The comparative analysis conducted in this study between *Arabidopsis* trichomes and *Nicotiana* LGTs and SGTs underscores their contrasting characteristics and relatively low similarity levels in expressed genes, thereby reaffirming previous conclusions. Concurrently, these novel observations broaden our understanding of trichome differentiation and provide insights into the distinctive features of trichomes across different plant species. It is anticipated that as single-cell technology is applied to more plant species with epidermal trichomes, we will gain deeper insights into the evolution of plant trichomes.

In conclusion, the comprehensive exploration of *Nicotiana* glandular trichomes and *Arabidopsis* leaf trichomes enriches our understanding of the biology of trichome development. The establishment of developmental trajectories, the metabolic comparisons among different types of GTs, and the comparative analysis with *Arabidopsis* trichomes collectively contribute to a more nuanced perspective on glandular trichome dynamics. These findings not only provide valuable insights into the development of specialized plant structures but also set the stage for future investigations, fostering a deeper exploration of glandular trichome biology.

### Limitations of the study

Of course, there are certain limitations of our study. First, we would like to enhance the marker genes differentiating between long- and short-stalked glandular trichomes by integrating more rigorous experimental procedures. If practicable, the mutants with the knockout of the target markers would significantly boost our understanding. At the same time, we propose a more detailed exploration of the comparative study between the epidermal hairs of *Arabidopsis* and the glandular trichomes of tobacco. This task could involve a more stringent investigation using comparative analyses at various time points. By delving further into these areas, it is expected to provide a deeper insight into this complex biological topic, and seamlessly integrate further study results with our existing findings.

## STAR★Methods

### Key resources table

**Table:** 

REAGENT or RESOURCE	SOURCE	IDENTIFIER
**Deposited data**		

raw and analyzed scRNA-seq datasets	this manuscript	PRJCA011350
raw data of 6-day-old *Arabidopsis* seedlings	Lee et al.^48^	GSE226097

**Critical commercial assays**		

Chromium Single Cell 3′ Library and Gel Bead Kit v3	10x Genomics	N/A
FISH *in situ* hybridization kit	Beyotime Biotechnology	N/A

**Oligonucleotides**		

see Table S13 for the list of primers used to generate FISH *in situ* hybridization kit	N/A	N/A

**Software and algorithms**		

R (version 4.0.2)	R software	http://www.R-project.org
Cell Ranger (version 6.0)	10x Genomics	https://support.10xgenomics.com/single-cell-gene-expression/software/overview/welcome
Seurat (version 4.0.0)	Hao et al.^64^	https://satijalab.org/seurat/index.html
ClusterVis (version 0.1.1)	Zhang^65^	https://github.com/junjunlab/ClusterGVis
clusterProfiler (version 3.16.1)	Yu et al.^66^	https://github.com/YuLab-SMU/clusterProfiler
AnnotationForge	Carlson et al.^67^	https://github.com/Bioconductor/AnnotationForge
org.At.tair.db	Carlson^68^	https://bioconductor.org/packages/release/data/annotation/html/org.At.tair.db.html
Monocle (version 2.28.0)	Qiu et al.^69^	https://github.com/cole-trapnell-lab/monocle-release?tab=readme-ov-file
ShinyCell (version 2.1)	Ouyang et al.^70^	https://github.com/SGDDNB/ShinyCell
ggplot2	Wickham et al.^71^	https://ggplot2.tidyverse.org/

### Resource availability

#### Lead contact

Further information and requests for resources and reagents should be directed to and will be fulfilled by the Lead Contact, Longjiang Fan (fanlj@zju.edu.cn).

#### Materials availability

This study did not generate new unique reagents.

#### Data and code availability

• The scRNA-seq data generated in this study have been deposited at NGDC database (https://ngdc.cncb.ac.cn/) and are publicly available as of the date of publication. Accession numbers are listed in the key resources table.
• This paper does not report original code.
• Any additional information required to reanalyze the data reported in this paper is available from the lead contact upon request.

### Experimental model and study participant details

#### Plant materials and growth conditions

Three accessions of *N*. *tabacum* with different composition of glandular trichomes (K326, B1000-1 and Basma) were used for leaf scRNA-seq experiments. The seeds were disinfected using 15% bleach solution, followed by germination in a growth chamber for 7 days. After germination, the seedlings were transplanted into soil and grown under long-day conditions at a temperature of 25°C during the day and 21 °C at night, with a light regime of 16 h of light (intensity of 80 mmol/m^2^/s) and 8 h of darkness. Leaf tissues were harvested after 40 days of growth.

### Method details

#### Single nucleus RNA extraction and processing

In this study, leaf samples were extracted from 40-day-old *N*. *tabacum* seedlings. Experimental analyses were conducted on leaf samples from three distinct types at the same developmental stage. Each leaf sample was meticulously collected and pooled from multiple plants to ensure the representativeness and reliability of the data. Nuclei were isolated from the leaf sample using the Singulator instrument (S2 Genomics) in iomics Biosciences Inc, Beijing. The instrument was primed with cold nuclei isolation and storage buffers (nuclei isolation buffers: 10 mM MES-KOH (pH 5.4), 10 mM NaCl, 10 mM KCl, 2.5 mM EDTA, 250 mM sucrose, 0.1 mM spermine, 0.5 mM spermidine, 1 mM DTT. Storage buffer: 20% glycerol, 20 mM HEPESKOH (pH 7.2), 5 mM MgCl2, 1 mM DTT.) and the sample was loaded into the cartridge and covered with the grinding cap. The “Nuclei_All_Tissues” protocol was used to isolate nuclei, after which the nuclei were centrifuged for 10 min and resuspended in buffer. The nuclei were stained with trypan blue and counted under a microscope, centrifuged again for 5 min and resuspended at the proper concentration for use in the Chromium Single Cell 3′ Library and Gel Bead Kit v3 (10x Genomics). Libraries were sequenced in one lane of a NextSeq 2000.

#### *Arabidopsis thaliana* seedling snRNA-seq data

Raw data of 6-day-old *Arabidopsis* seedlings were download from GEO of NCBI (accession number GSE226097).^48^ The snRNA-seq reads were aligned to the reference genome Araport11, downloaded from the TAIR database (https://www.arabidopsis.org/).

#### snRNA-seq data analysis

The Cell Ranger pipeline (version 6.0) provided by 10x Genomics was used to map reads to the *N. tabacum* reference genome K326 and its annotated gene set^72^ downloaded from the Solanaceae Genomics Network at https://solgenomics.net/organism/Nicotiana_tabacum/genome and https://solgenomics.net/ftp/genomes/Nicotiana_tabacum/sierro_et_al_2014/annotation/. Given the issue of gene identification numbers, we have included more detailed annotation information in correspondence (Table S1). All downstream single-cell analyses were performed using Seurat (v4.0.0).^64^ In brief, the gene-cell matrices were loaded into the Seurat package, which is implemented in R (v. 4.0.2). To remove low quality cells, we filtered out the cells with unique gene counts fewer than 200. Apart from adhering to the minimum gene criteria, the cellular data was further refined by setting the threshold for mitochondrial percentage at 0.5%, and for chloroplasts at 5% (Figure S2). The genes expressed in at least three single cells were kept. Read count normalization, variable feature detection (nfeatures = 2000), scaling, UMAP (ndim = 50), and differential expression were computed as described in the Seurat package. Clustering was performed by the Louvain algorithm (resolution = 0.5). Finally, the FindAllMarkers function was used to identify marker genes that were up-regulated in each cluster versus all other cells (average FC ≥ 0.25 plus maximum adjusted *P*-value ≤0.05). Cell types were characterized by a combination of known markers and GO enrichment result based on *de novo* cluster markers (detailed annotation information see Table S2). The ClusterVis software was utilized to display marker gene expression.^65^

#### GO enrichment analysis

Enriched GO terms associated with cluster-specific expressed genes were identified by clusterProfiler (version 3.16.1)^66^ compareCluster and gofilter (level = 3) with the following parameters fun = "enrichGO", OrgDb = "org.Ntabacum.e.g.,.db" (established by the R package AnnotationForge), ont = "ALL", pAdjustMethod = "BH", pvalueCutoff = 0.05, qvalueCutoff = 0.05.

#### Pseudotime analysis

To mitigate potential analysis errors arising from inter-type differences, we utilized data from one type (K326) for the analyses. K326 is a widely cultivated variety or type of tobacco with a substantial researches on it as a model plant. Its genome has been sequenced^72^ and used as a reference for *Nicotiana* studies. The well-annotated gene set of K326 was used in this study. We processed our analyses with the Monocle package (version 2.28.0), a tool extensively used for single-cell RNA sequencing data to model cell differentiation and thereby determine cell fate. The first step was to calculate the expression variance of each gene across cells by running 'dispersionTable'. Variable genes whose average expression level (represented by the 'mean expression' parameter) exceeded a predetermined threshold were selected for defining a developmental trajectory or 'pseudo-time'. Next, using the feature 'max_components = 2, method = DDRTree', we reduced the dimensionality of the data. This allowed us to depict the expression data within a two-dimensional space, which is more manageable and interpretable. Now, with cells plotted across a two-dimensional space, the transition of cells from one state to another could be carefully tracked. We carried this out using the 'orderCells' function in Monocle, which allowed the ordering of cells in pseudo-time. We then plotted the cell trajectory using 'plot_cell_trajectory' in Monocle, providing a visual representation of the developmental journey of each cell as it transforms from one state to another. Furthermore, we employed BEAM (Branched Expression Analysis Modeling) to analyze the pseudo-time-dependent or branch-dependent genes. Genes that were significantly branch dependent were visualized using the 'plot_genes_branched_heatmap' function. Lastly, the data used for plotting heatmaps were subjected to GO enrichment analysis, which enabled us to identify significant functional themes and biological processes within our gene sets.

#### Analysis of *arabidopsis* epidermal trichome microarray data

The microarray data of *Arabidopsis* epidermal trichome were downloaded from the EMBL-EBI database (E-MEXP-2008). The affy package in R was employed for the data processing in which the 'expresso' function was used to carry out the entire procedure: firstly, background correction was performed using the bgcorrect.method = "rma" argument to eliminate potential noise or interference; secondly, data normalization was carried out with the normalize.method = "quantiles" argument to ensure data consistency and comparability; next, the pmcorrect.method = "pmonly" parameter was responsible for adjusting the PM after RMA correction; finally, the summary.method = "medianpolish" argument was used to summarise the normalized data. The gene expression heatmap was then drawn using the pheatmap package in R.

#### Construction of interactive shiny web pages

Interactive Shiny-based web applications were created by integrating Seurat integrated objects into ShinyCell v2.1^70^ to facilitate data exploration.

#### *In situ* hybridization

*N. tabacum* leaves were fixed using RNase free 4% paraformaldehyde at room temperature. Antisense probes were designed for each target gene in our study, assuring the specificity of our experiment although no sense probes were used. We employed a specific nematode sequence as a negative control for detecting non-specific hybridization. Since this particular sequence theoretically doesn’t exist in our tobacco samples, any resulting signal would suggest the presence of non-specific hybridization or potential procedural errors, thereby validating the accuracy of our results. The labeled DNA probes (Table S13) were hybridized with the samples using the FISH *in situ* hybridization kit (Beyotime Biotechnology, Catalog No: C007). After completion of the hybridization reaction, cell nuclei were stained with DAPI. Fluorescence microscopy was used to observe the nuclei of leaf cells at a wavelength of 340 nm, and the hybridization signals were observed at a wavelength of 552 nm.

### Quantification and statistical analysis

Bioinformatic analysis was described in the method details section. All statistical analyses were processed on R (v4.0.2) software, and *p* value < 0.05 was considered statistically significant.

## Supplemental information

Document S1. Figures S1–S14 and Tables S2, S11 and S13

Table S1. Gene annotation information, related to STAR method

Table S3. Cell type-specific differential genes, related to Figure 2

Table S4. GO enrichment results of cell types, related to Figure 2

Table S5. LGTs-specific genes, related to Figure 2

Table S6. SGTs-specific genes, related to Figure 2

Table S7. LGTs branch-point differential genes, related to Figure 3

Table S8. SGTs branch-point differential genes, related to Figure 3

Table S9. Differential genes in N. tabacum LGTs, related to Figure 4

Table S10. Differential genes in N. tabacum SGTs, related to Figure 4

Table S12. Arabidopsis trichomes branch-point differential genes, related to Figure 5
