# Supplementary material for: Single-cell landscape of long and short glandular trichomes in Nicotiana tabacum leaves
Source: iScience. 2024 Aug 3;27(9):110650. doi: 10.1016/j.isci.2024.110650 (PMC11382123; doi:10.1016/j.isci.2024.110650)
Supplement: Document S1. Figures S1–S14 and Tables S2, S11 and S13 [file mmc1.pdf]

iScience, Volume *27*

Supplemental information

Single-cell landscape of long and short glandular trichomes in *Nicotiana tabacum* leaves

Hongyu Chen, Xiaohan Li, Qing Cheng, Nianmin Shang, Zhijun Tong, Qinjie Chu, Chuyu Ye, Xiner Shen, Qian-Hao Zhu, Bingguang Xiao, and Longjiang Fan

Supplemental Materials

Table S2 List of marker genes for cell type annotation, related to Figure 1.

Cell type	Gene name	Orthologs	Gene symbol	Annotation
epidermis	gene_68281	AT1G72970	*NtHTH*	Glucose-methanol-choline (GMC) oxidoreductase family protein
epidermis	gene_36872	AT1G72970	*NtHTH*	Glucose-methanol-choline (GMC) oxidoreductase family protein
epidermis	gene_35601	AT5G23940	*NtPEL3*	HXXXD-type acyl-transferase family protein
G2M phase	gene_48930	AT3G25980	*NtMAD2*	hydroxycinnamoyl-CoA shikimate/quinate hydroxycinnamoyl transferase
G2M phase	gene_6872	AT2G26760	*NtCYCB1*	Cyclin family protein
G2M phase	gene_23349	AT4G31840	*NtENODL15*	early nodulin-like protein 15
guard cell	gene_71917	AT3G24140	*NtFMA*	basic helix-loop-helix (bHLH) DNA-binding superfamily protein
guard cell	gene_41667	AT1G33811	*NtGGL7*	GDSL-like Lipase/Acylhydrolase superfamily protein
guard cell	gene_77033	AT1G33811	*NtGGL7*	GDSL-like Lipase/Acylhydrolase superfamily protein
mesophyll	gene_32951	AT3G01500	*NtCA1*	carbonic anhydrase 1
mesophyll	gene_59414	AT3G01500	*NtCA1*	carbonic anhydrase 1
mesophyll	gene_12634	AT1G70760	*NtNdhL*	inorganic carbon transport protein-related
phloem	gene_62938	AT1G79430	*NtAPL*	Homeodomain-like superfamily protein
phloem	gene_81249	AT1G79430	*NtAPL*	Homeodomain-like superfamily protein
phloem	gene_74664	AT2G03500	*NtEFM*	Homeodomain-like superfamily protein
G1/S phase	gene_65414	AT5G59970	*AT5G59970*	Histone superfamily protein
G1/S phase	gene_24656	AT5G02560	*NtHTA12*	histone H2A 12
G1/S phase	gene_44765	AT5G65360	*NtHTR1*	Histone superfamily protein

vascular	gene_14488	AT3G54560	*NtHTA11*	histone H2A 11
vascular	gene_61622	AT3G54560	*NtHTA11*	histone H2A 11
vascular	gene_77408	AT3G54560	*NtHTA11*	histone H2A 11
xylem	gene_80772	AT2G21050	*NtLAX2*	like AUXIN RESISTANT 2
xylem	gene_814	AT2G21050	*NtLAX2*	sulfur E2
xylem	gene_7817	AT2G21050	*NtLAX2*	like AUXIN RESISTANT 2
glandular trichome	gene_51803	AT5G23960	*NtTPS21*	terpene synthase 21(from tobacco)
glandular trichome	gene_56342	AT2G23230	*NtPRORP1*	Terpenoid cyclases/Protein prenyltransferases superfamily protein(from tobacco)
glandular trichome	gene_28845	AT4G02780	*NtCPS2*	Terpenoid cyclases/Protein prenyltransferases superfamily protein(from tobacco)
glandular trichome	gene_63422	AT3G26300	*NtCYP71D16*	cytochrome P450, family 71(from tobacco)

Table S11 The highly expressed genes that are specifically common between the Arabidopsis epidermal trichome and tobacco long and short glandular trichome, related to Figure 5.

Arabidopsis gene	Tobacco gene
AT4G39260	gene_42071
AT2G47240	gene_11844;gene_17104
AT1G72970	gene_36872
AT1G27950	gene_18123
AT5G25880	gene_19355
AT1G79750	gene_19355
AT4G13840	gene_20149
AT2G34680	gene_24130;gene_27814
AT1G49430	gene_26757
AT3G25840	gene_28443
AT2G24280	gene_34670
AT2G01100	gene_35070
AT5G08630	gene_394
AT2G41430	gene_41759
AT3G44340	gene_51684
AT3G14310	gene_55675
AT2G21660	gene_60324
AT1G20440	gene_61506
AT1G20620	gene_68512
AT4G37870	gene_7839

Table S13 The highly expressed genes that are specifically common between the Arabidopsis epidermal trichome and tobacco long and short glandular trichome, related to STAR Methods.

Gene	Probe sequence
gene_67244	5’-CTCGTGTCAATGATGCCAATGACGCACTAATGGATTGGAGCTGTT-3’
gene_50725	5’-CAATTTGCTGACTGGCGGGAGCACGCCAAATGCATCACATGCTAG-3’
gene_35261	5’-CTTGATATCACATTTGTTGCTCCACACCGCAATTGCACTGATGCA-3’
gene_37943	5’-CAGCTCAGGCTCAGCTCTTCAAGTTAGCCAATGAGGGTCGGCTC-3’
gene_10705	5’- CATGGATTCAAGTTTCAGCTCATATGAAATTGACTCAACCTTGTCC-3’
gene_65106	5’- CAACATGGATAGCTTTCGAACATACATTTCAAGATATTATTGGCC-3’

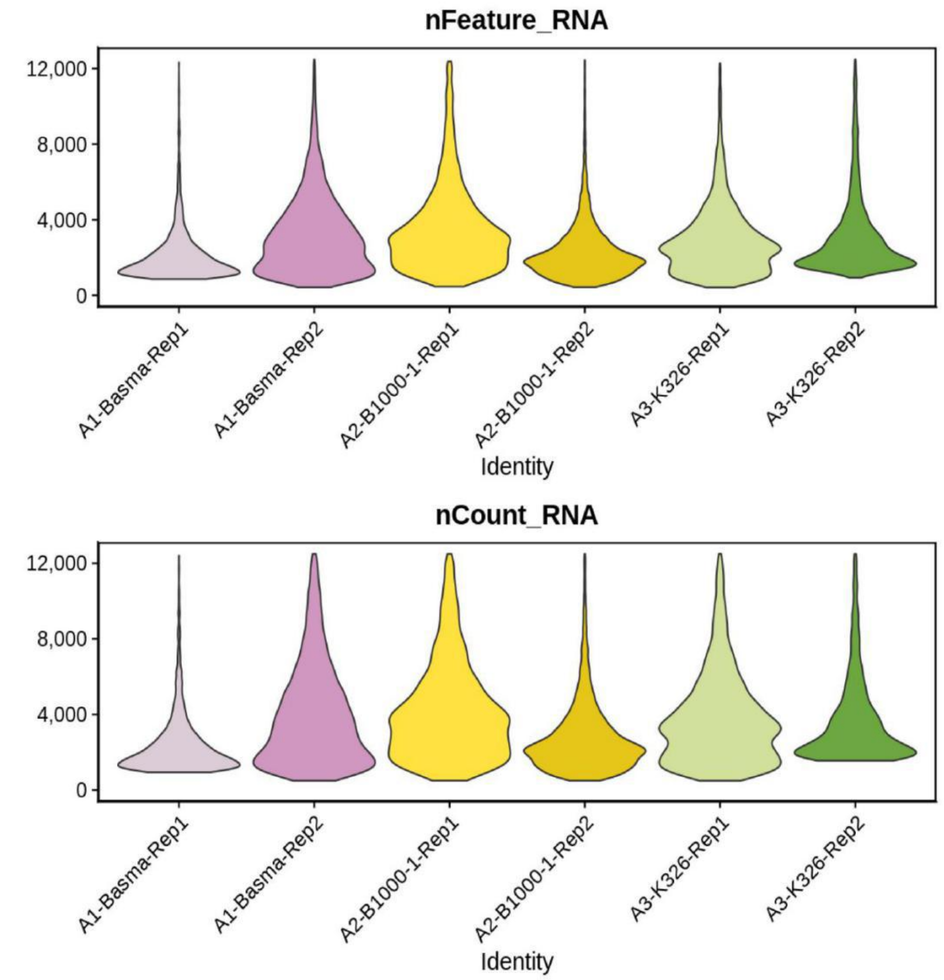

Figure S1. Violin plots displaying the detected feature number (nFeature_RNA) and the read counts (nCount_RNA) in each sample, related to Figure 1.

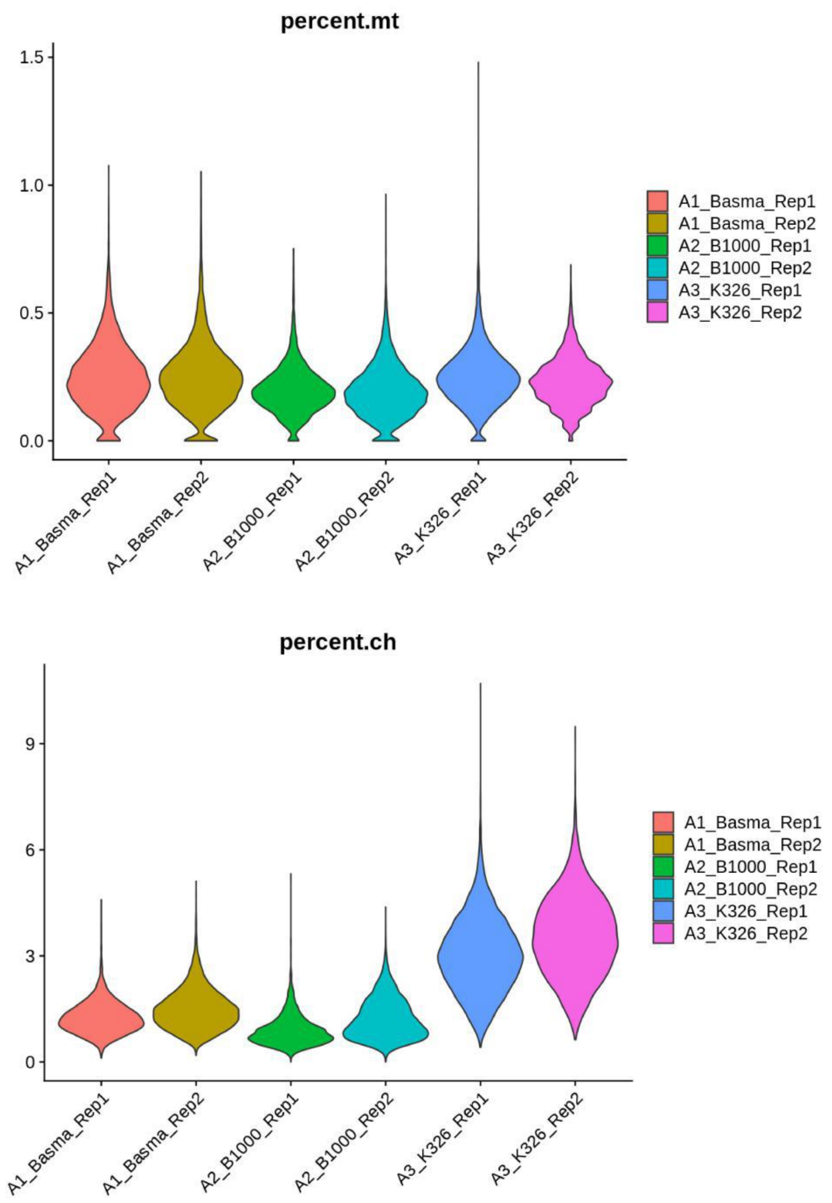

Figure S2. The violin plot illustrates the distribution of gene expression in mitochondria (percent.mt) and chloroplasts (percent.ch) across all samples, related to Figure 1.

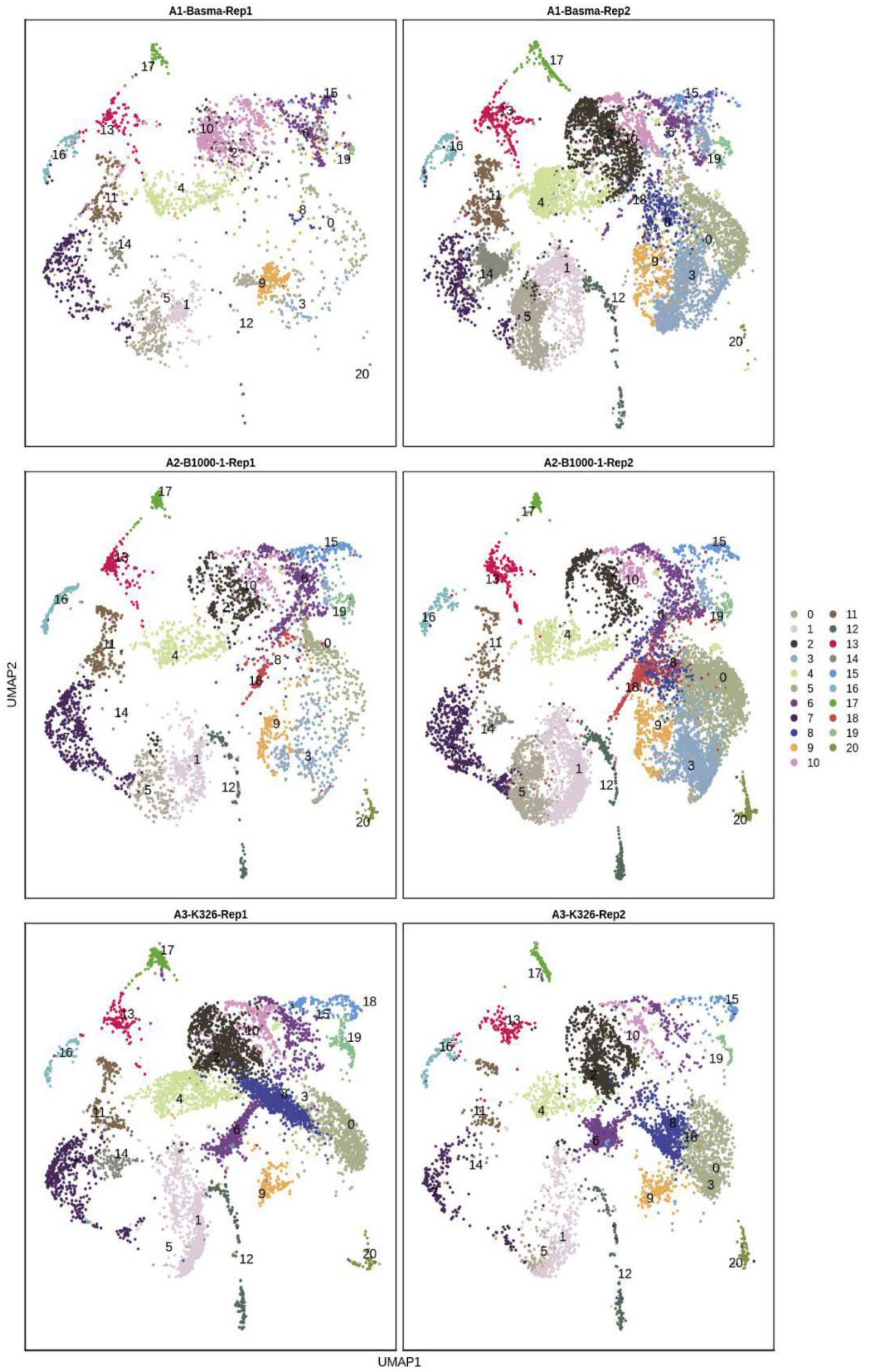

Figure S3. UMAP plots of the 6 samples from 3 Nicotiana accessions, demonstrating both the robust reproducibility between the two samples of the same accessions and the consistency in the number of cell types identified across different accessions, related to Figure 1.

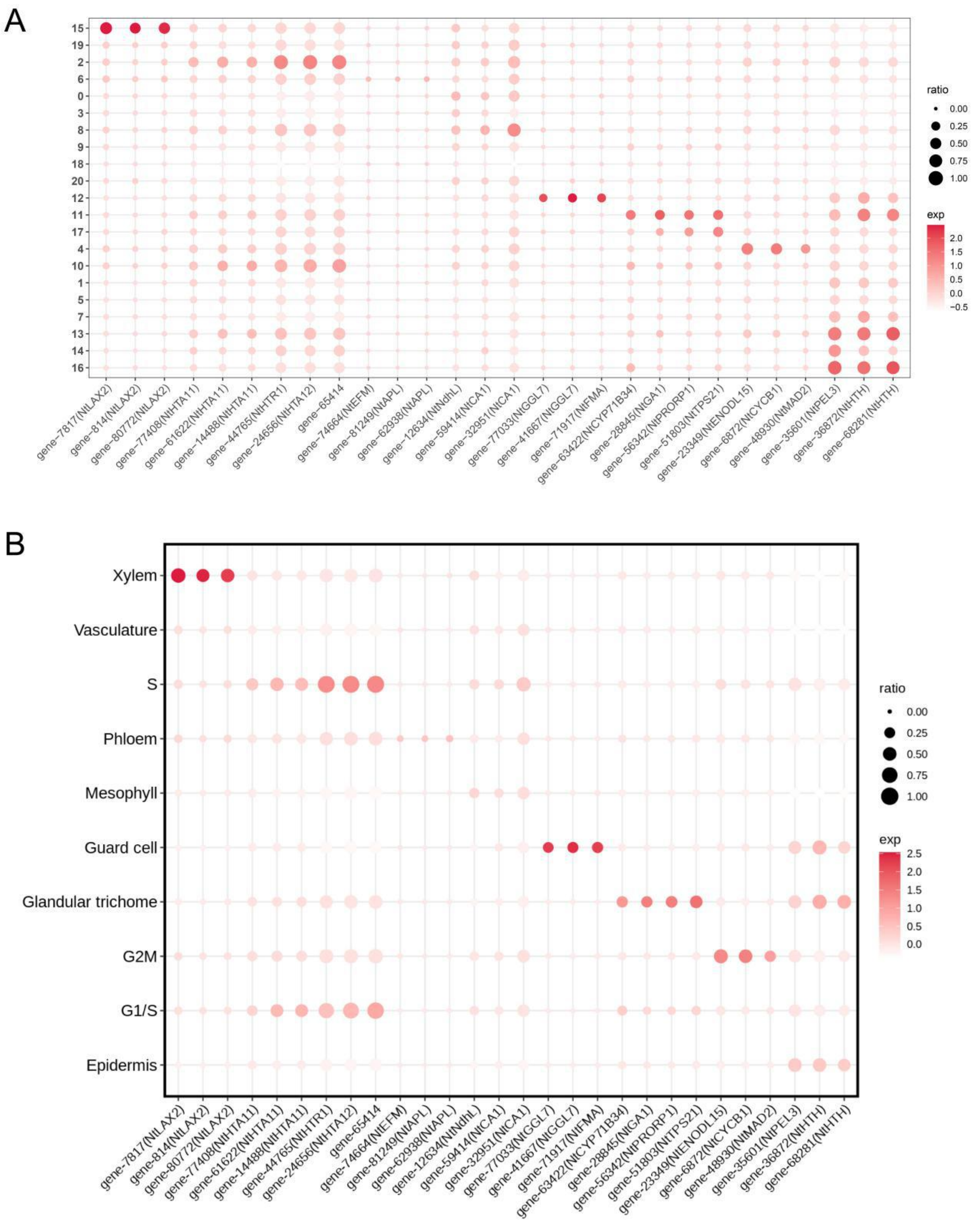

Figure S4. Expression patterns of marker genes for cell types are displayed, related to Figure 1. (A) A dot plot illustrates the expression of leaf cell type marker genes across cell clusters. (B) A dot plot shows the expression of leaf cell type marker genes within various cell groups.

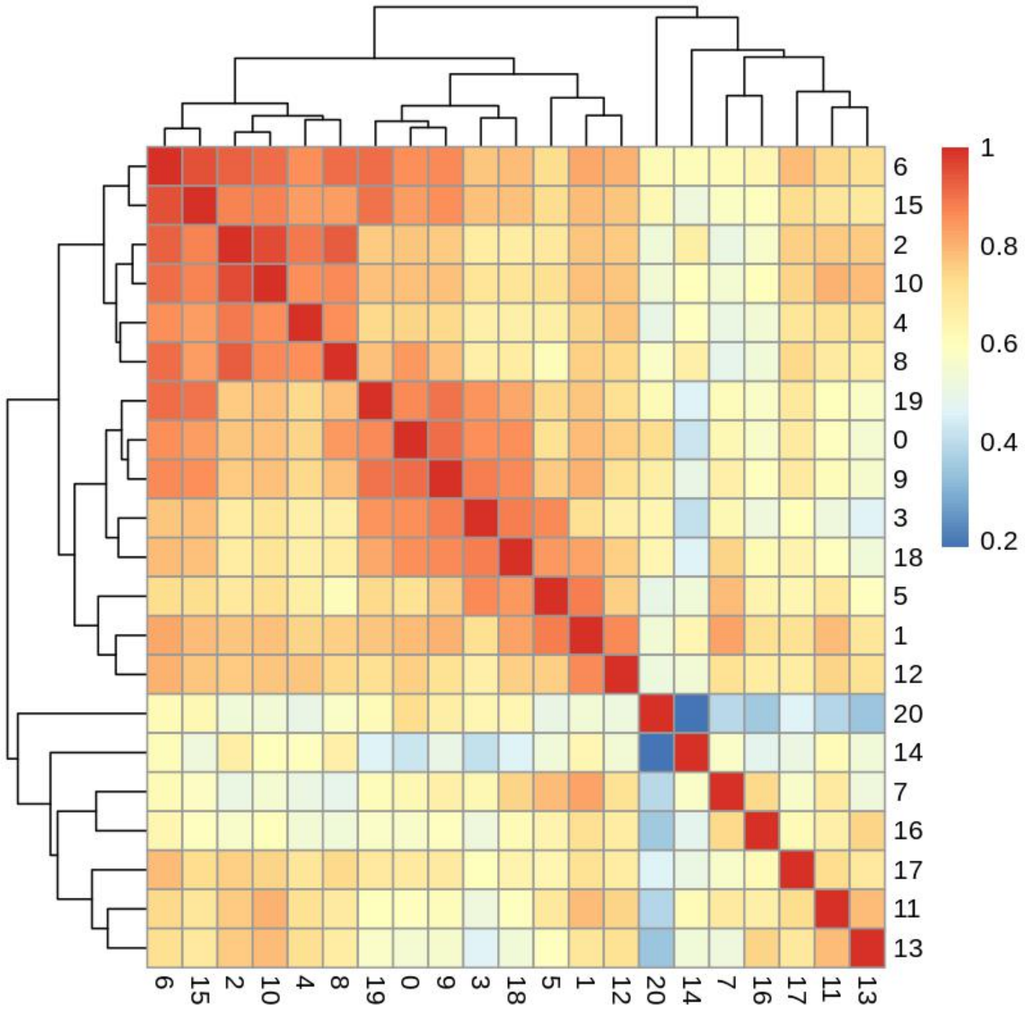

Figure S5. Correlation heatmap displaying the inter-cluster relationships, related to Figure 1.

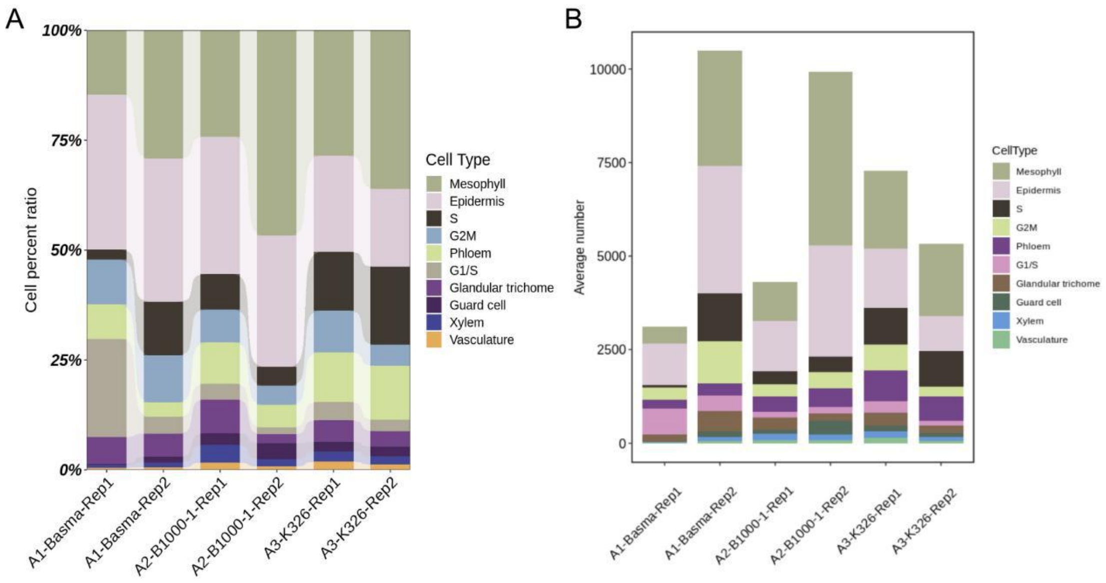

Figure S6. The distribution of various cell types among different accessions and samples is examined, related to Figure 1. (A) Bar chart illustrating the proportions of cell types among different accessions and samples. (B) Bar chart illustrating the numbers of different cell types among various accessions and samples.

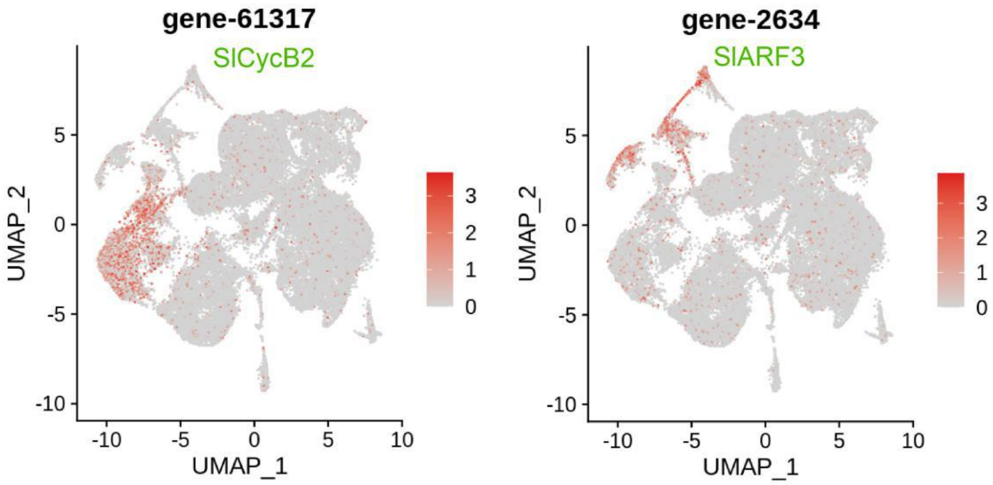

Figure S7. Expression of tomato trichome marker genes in tobacco leaves, related to Figure 1.

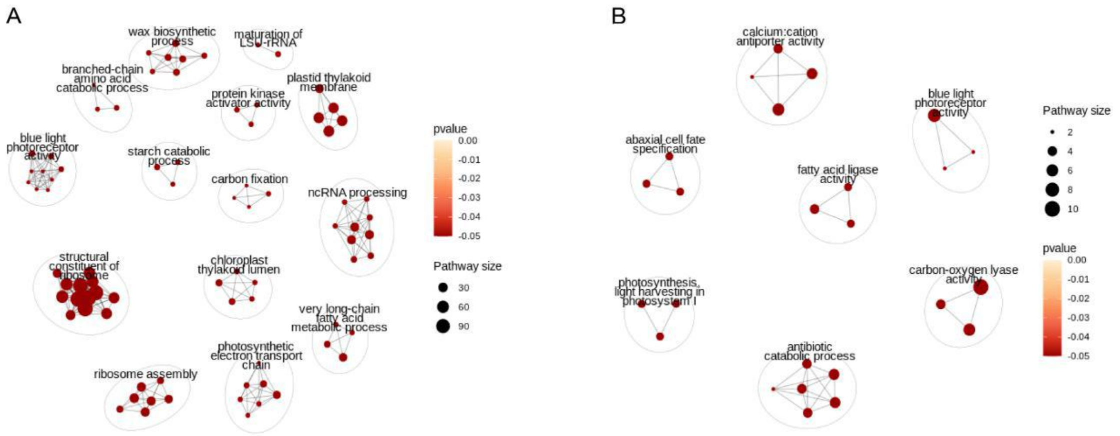

Figure S8. The functional enrichment results of GT-specific expression genes are presented, related to Figure 1. (A) A network diagram displays LGT-specifically enriched GO terms. (B) A network diagram displays SGT-specifically enriched GO terms.

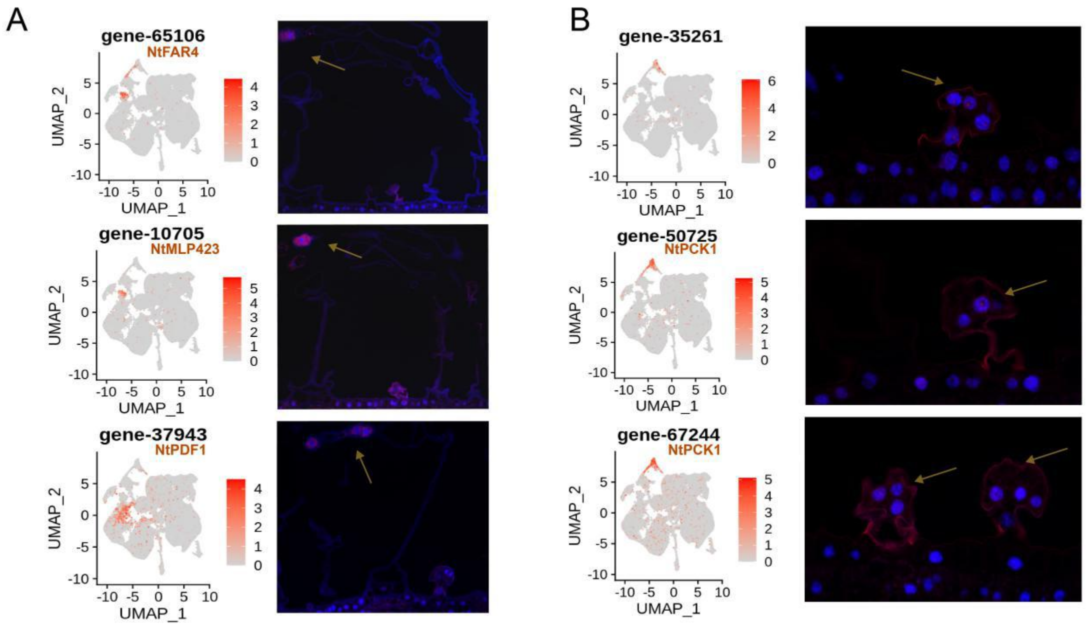

Figure S9. Expression profiles of the top three GT-specific genes in UMAP clusters, with in situ hybridization results on the right, related to Figure 1. (A) Display the specifically expressed genes identified in LGT. (B) Display the specifically expressed genes identified in SGT.

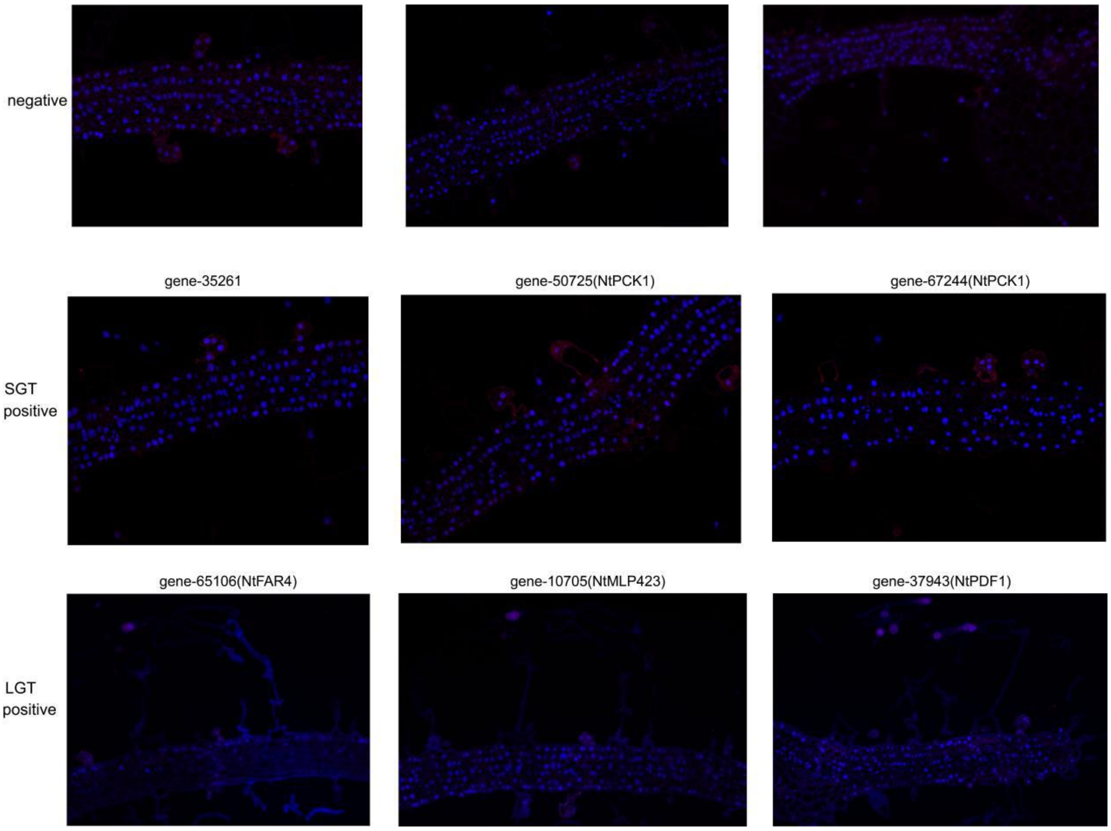

Figure S10. In situ hybridization images of tobacco glandular trichome marker genes, where 'negative' denotes negative controls and 'positive' represents positive results. 'LGT' corresponds to Long Glandular Trichome, and 'SGT' refers to Short Glandular Trichome, related to Figure 1.

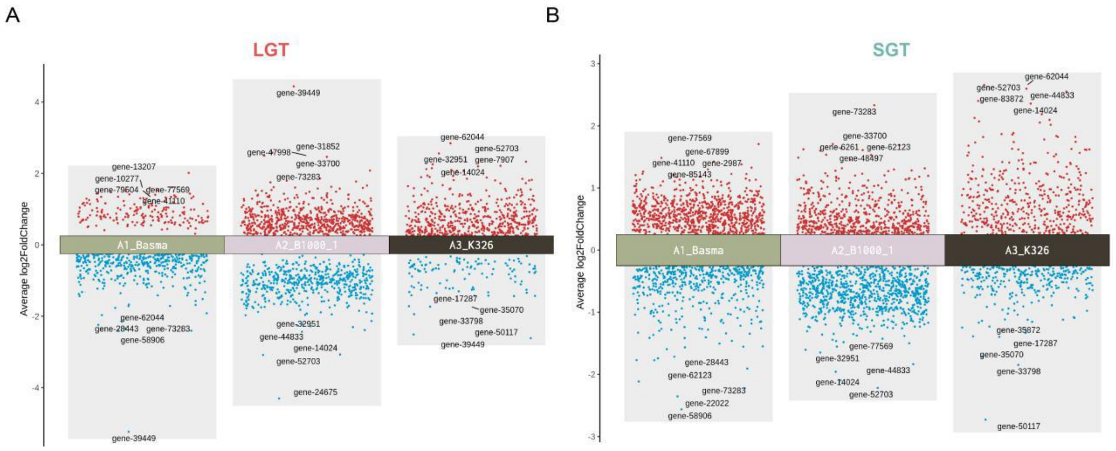

Figure S11. Genes specifically expressed in trichomes of different tobacco accessions, related to Figure 4. (A) Volcano plot displaying LGTs genes specifically expressed in different N. tabacum accessions. (B) Volcano plot displaying SGTs genes specifically expressed in different N. tabacum accessions.

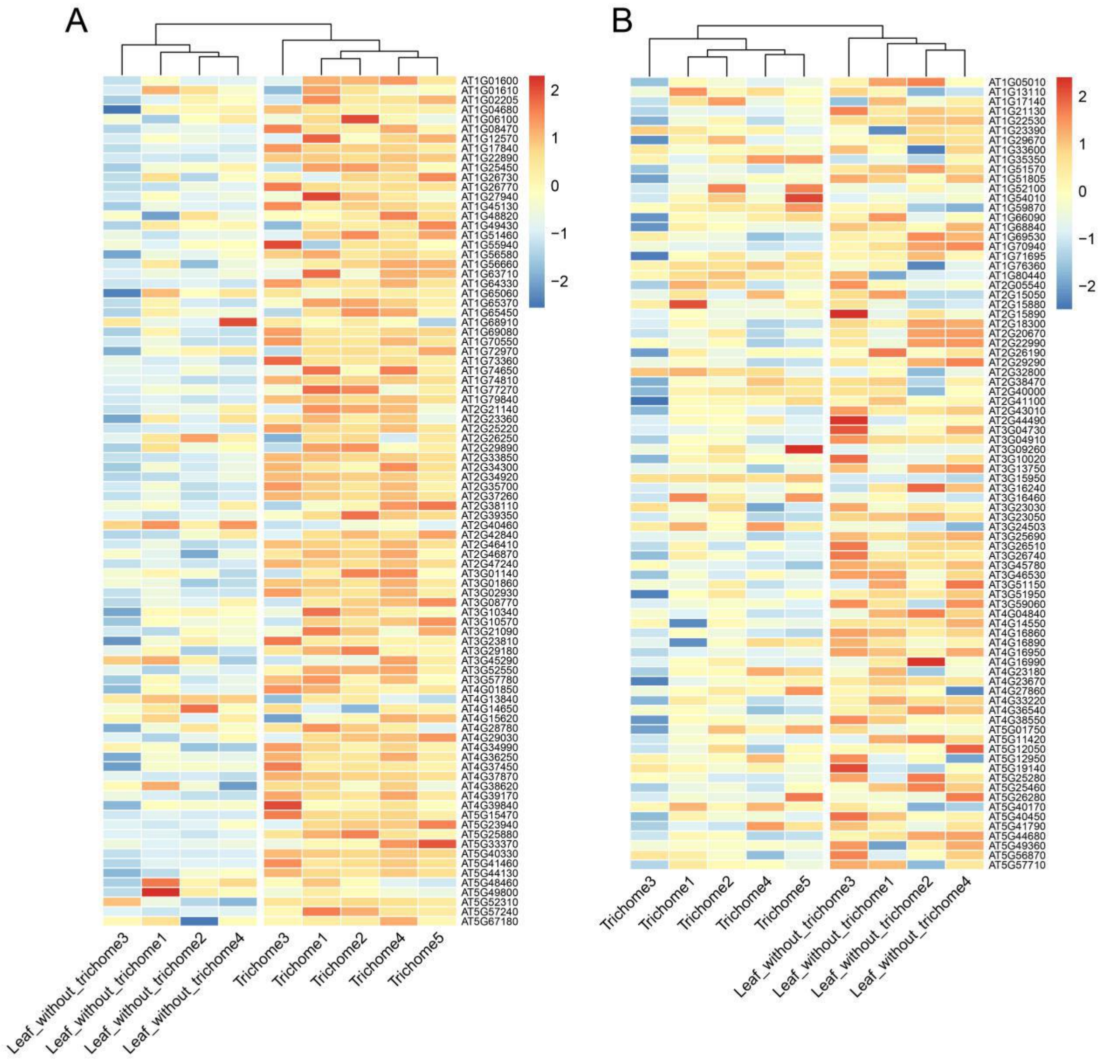

Figure S12. The heatmap affirms that the trichome-specific genes identified from single-cell data in Arabidopsis are also highly expressed in the corresponding microarray transcriptomic data, related to Figure 5.

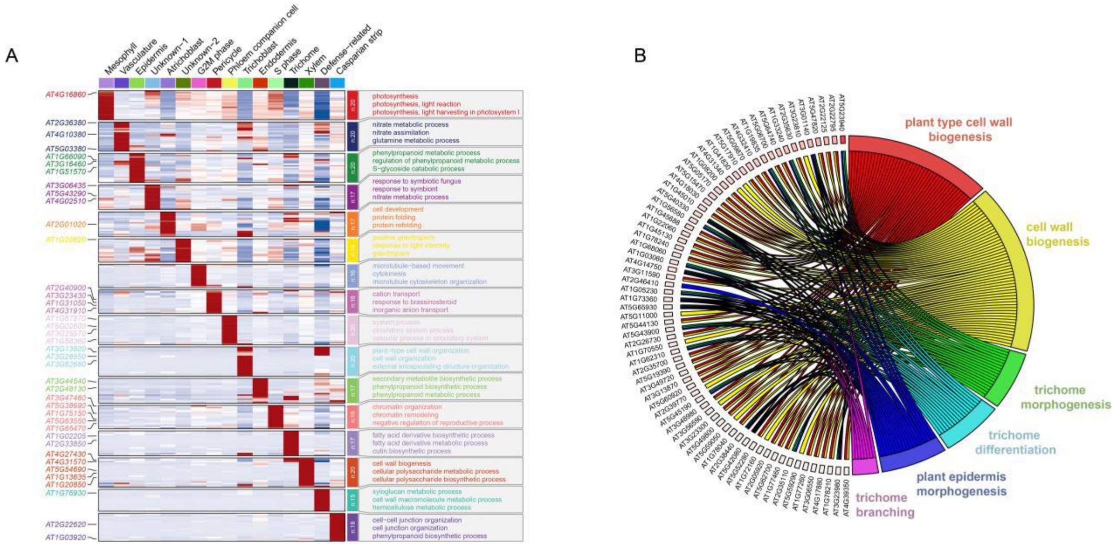

Figure S13. Specifically expressed genes in different cell types and their Gene Ontology (GO) enrichment results, related to Figure 5. (A)Heat map showing the identified cell type-specific differentially expressed genes, with the representative enriched functional terms displayed on the right. The bar chart in the middle illustrates the number of enriched genes. (B) Circular plot depicting the relationship between the shared specific genes in epidermal trichomes of *Arabidopsis*, LGTs and SGTs of *N. tabacum*, along with the associated GO terms.

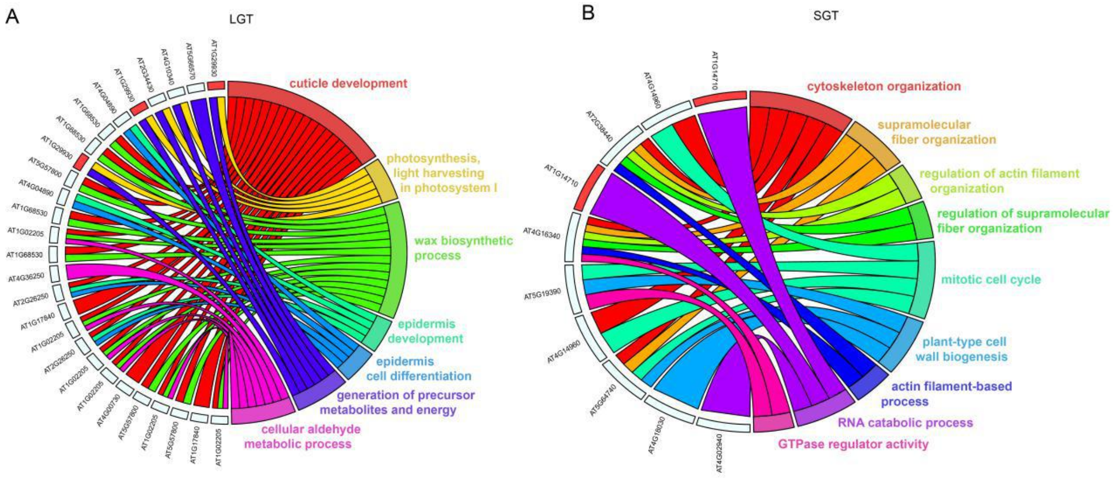

Figure S14. GO enrichment analysis of genes specifically expressed in long and short glandular trichomes of tobacco, related to Figure 5. (A) Circular plot depicting the relationship between the shared specific genes in epidermal trichomes of *Arabidopsis* and LGTs of *N. tabacum*, along with the corresponding GO terms. (B) Circular plot depicting the relationship between the shared specific genes in epidermal trichomes of *Arabidopsis* and SGTs of *N. tabacum*, along with the corresponding GO terms.
